# Multi-stage volcanic island flank collapses with coeval explosive caldera-forming eruptions

**DOI:** 10.1038/s41598-018-19285-2

**Published:** 2018-01-18

**Authors:** James E. Hunt, Michael Cassidy, Peter J. Talling

**Affiliations:** 1 National Oceanography Centre, Southampton, European Way, Southampton, SO143ZH UK; 2 Department of Earth Sciences, University of Oxford, South Parks Road, Oxford, OX13AN UK; 3 Departments of Earth Sciences and Geography, Durham University, Durham, DH1 3LE UK

## Abstract

Volcanic flank collapses and explosive eruptions are among the largest and most destructive processes on Earth. Events at Mount St. Helens in May 1980 demonstrated how a relatively small (<5 km^3^) flank collapse on a terrestrial volcano could immediately precede a devastating eruption. The lateral collapse of volcanic island flanks, such as in the Canary Islands, can be far larger (>300 km^3^), but can also occur in complex multiple stages. Here, we show that multistage retrogressive landslides on Tenerife triggered explosive caldera-forming eruptions, including the Diego Hernandez, Guajara and Ucanca caldera eruptions. Geochemical analyses were performed on volcanic glasses recovered from marine sedimentary deposits, called turbidites, associated with each individual stage of each multistage landslide. These analyses indicate only the lattermost stages of subaerial flank failure contain materials originating from respective coeval explosive eruption, suggesting that initial more voluminous submarine stages of multi-stage flank collapse induce these aforementioned explosive eruption. Furthermore, there are extended time lags identified between the individual stages of multi-stage collapse, and thus an extended time lag between the initial submarine stages of failure and the onset of subsequent explosive eruption. This time lag succeeding landslide-generated static decompression has implications for the response of magmatic systems to un-roofing and poses a significant implication for ocean island volcanism and civil emergency planning.

## Introduction

Volcanic island landslides, their associated tsunamis, and volcanic eruptions pose significant natural hazards to both life and infrastructure. Lateral flank collapse of shield volcanoes can yield prodigious landslides with volumes in excess of 300 km^3^, such as those in the Hawaiian, Canarian, Cape Verdean and Reunion archipelagos^1–8^. These are among the largest mass movements on Earth, whose size is far larger than any subaerial landslide^9^. Such voluminous landslides are especially hazardous because they may cause high-amplitude tsunamis on entering the ocean and also be directly associated with major volcanic eruptions^10–14^.

Direct observations of volcano flank collapse on land have shown that they can be immediately succeeded by major explosive eruptions. For instance, events at Mount St Helens in 1980 showed how ascent of magma and emplacement of a crytodome caused the edifice to inflate, oversteepen and become unstable^15,16^. On May 18 1980 at 08:22:11 local time, a 5.1 M_w_ earthquake related to magma intrusion occurred and triggered a single-stage flank failure ten seconds later comprising two slide blocks^17,18^. The resulting landslide was followed after thirty seconds by a devastating series of explosive eruptions including a lateral blast that took fifty-eight lives, cost over a billion US$ in damages, and resulted in unquantified potential respiratory health risks to local residents involved^15,16,18,19^. In addition to the immediately post-collapse lateral blast at Mount St Helens, intense explosive activity continued for nine hours after the initial failure; this was followed by further explosive activity on 25 May, 12 June, 22 July, 7 August and 16–18 October 1980. The later episodes of activity in this sequence punctuated lava dome growth accompanied by frequent minor explosions^17^.

Mount St Helens is not an isolated example. Well constrained chronologies of the Harimkotan 1933 Bezymianny 1956 and Shiveluch 1964 demonstrate that initial flank collapses were followed by explosive eruptions^20^. Indeed, the 1964 event at Shiveluch demonstrates that an initial collapse was followed first by a phreatic explosion and then an explosive Plinian eruption^20^. Analysis of older debris avalanches at Shiveluch indicates this scenario may have been common in the past^21–23^. However, many stratovolcano lateral collapses do not show evidence of coincidental lateral blasts or other types of explosive eruption^24,25^. Thus, terrestrial volcanoes do not show a consistent coincidence of flank collapse and eruptions, and so there are issues with invoking a cause-and-effect relationship. However, past events on Tenerife, in the Canary Islands, may elucidate the range of relationships between flank collapses and large explosive eruptions.

**Figure 1:**
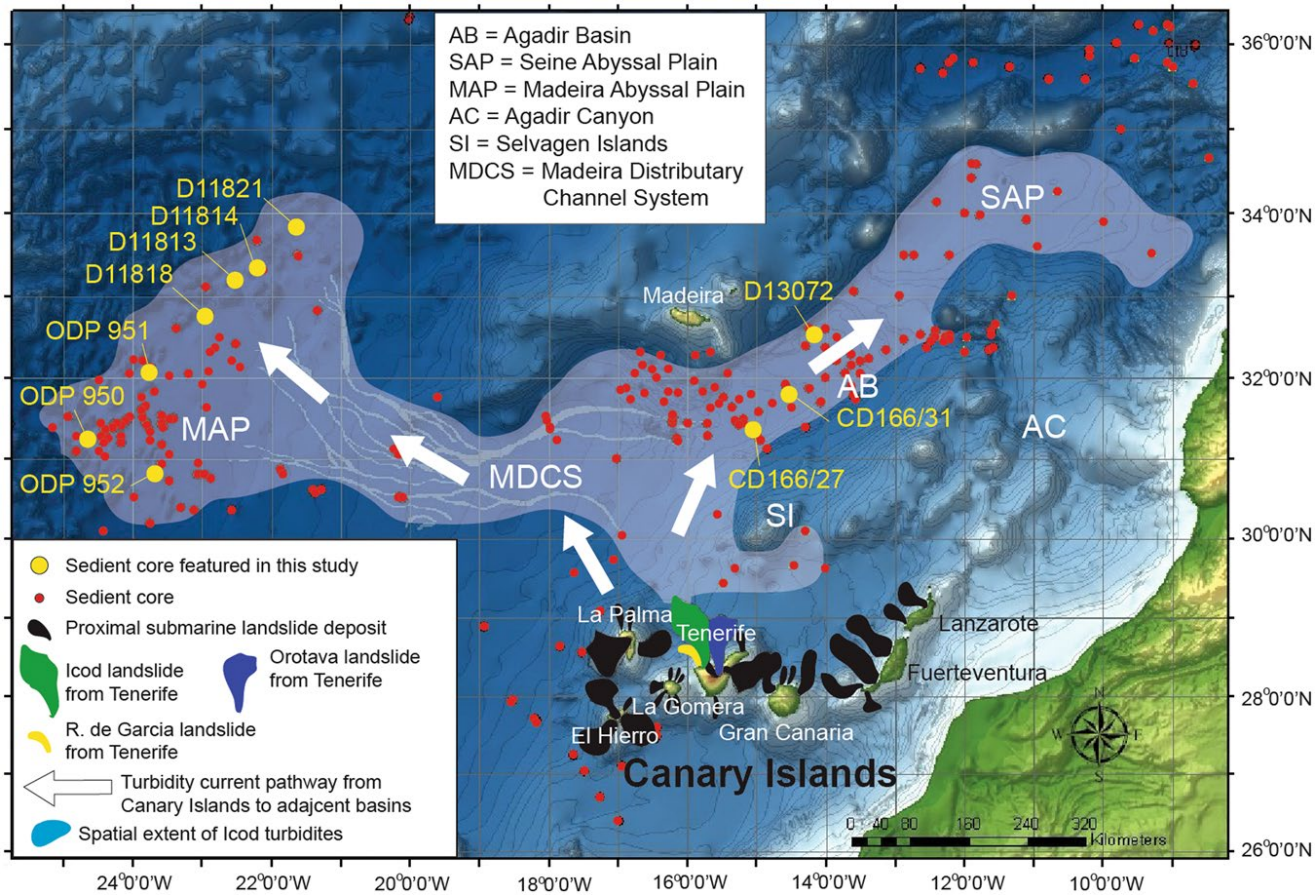
Map of the Canary Islands and the Moroccan Turbidite System. Canary Islands debris avalanche deposits shown in black, while Icod deposit is in dark green, the Orotava deposit in dark blue, Roques de Garcia in red, and Cumbre Neuva in yellow. Pale overlay signifies the spatial distribution of the sediment gravity flow deposits associated with the Icod landslide from Northern Tenerife. Sediment core locations are shown as red circles, while cores referenced in this contribution are in yellow and labelled (including ODP Sites 950, 951 and 952). Abbreviations are: AC = Agadir Canyon, AB = Agadir Basin, MAP = Madeira Abyssal Plain, SAP = Seine Abyssal Plain, MDCS = Madeira Distributary Channels, and CBR = Casablanca Ridge. Canary Islands landslides Icod (brown), Orotava (purple) and Rogues de Garcia (red) from Tenerife and Cumbre Nueva (grey). May also shows the area extent of the turbidites associated with the Icod landslide. Map was generated using *ArcGIS* 10.1 software based upon bathymetric and digital elevation data from *GEBCO*.

**Figure 2:**
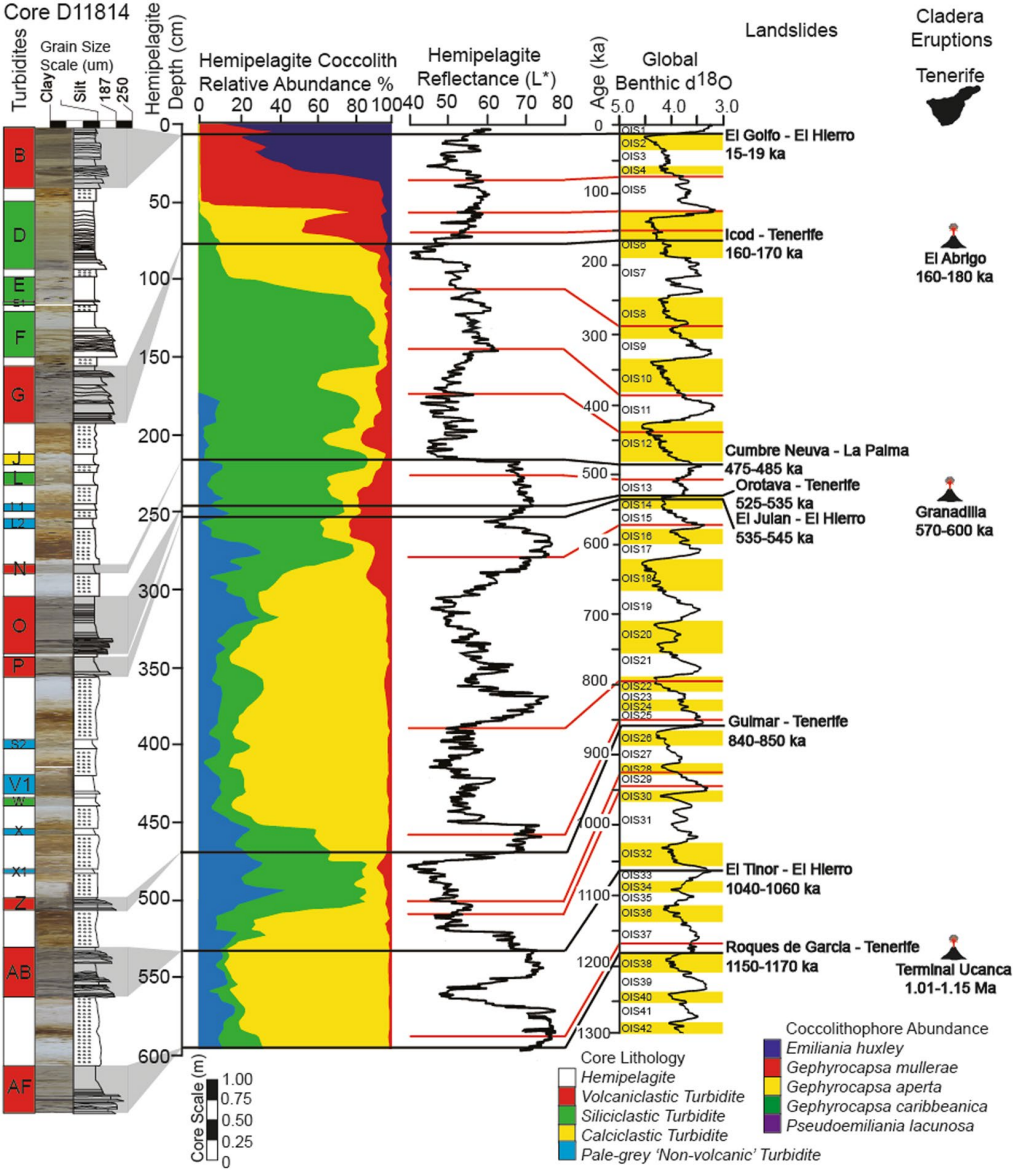
Timing of flank collapses from Northern Tenerife and caldera-forming eruptions. Volcaniclastic turbidites from the Madeira Abyssal Plain recorded at site D11814 (location on Fig. 1). Biostratigraphic and photospectral data from Hunt *et al*.^7^. Timing of caldera-forming eruptions from Marti *et al*.^35^. Further information regarding the timing of landslides and caldera eruptions and the nature of the deposits are documented in supplement 1.

The Canary Islands is among the most active volcanic archipelagos. Indeed, Tenerife is one of the world’s largest active volcanoes having experienced numerous Plinian eruptions and caldera-collapses, many of which have ejected volumes up to fifty times greater than the 1980 Mount St Helens eruption^26–28^. Furthermore, the Canary Islands, and in particular Tenerife, have incurred numerous large landslides over the last seven million years (roughly every 150 to 250 kyr) with volumes often over 300 km^3^ (Fig. 1)^6–8^. There is growing evidence that suggests explosive eruptions and mass wasting processes have a cause-and-effect relationship in ocean island settings^7,12,29–34^; whereas some other terrestrial volcanoes show no such relationship^9,24,25^. During the development of the Canadas Upper Group (1.56 Ma to 0.17 Ma) on Tenerife, in the Western Canary Islands, the Canadas volcano underwent three distinctive phases of growth and destruction, each ending with both a caldera eruption and a coeval lateral flank collapse (Fig. 2)^7,8,29–35^. Thus Tenerife presents an excellent opportunity to examine the relationships between these two destructive processes.

Previous studies postulate that caldera-forming and similar explosive eruptions may cause ground accelerations that trigger flank collapse^33,34^. For example, the 2002 explosive eruption of Stromboli Volcano caused a sequence of subsequent tsunamigenic landslides^36^. The involvement of caldera-forming eruptions on ocean islands influencing flank collapse is postulated with the specific reference to the formation of the Icod and Orotava Valleys on Tenerife^31–34^. However, preliminary evidence from distal marine deposits of the Icod landslide (165 ka) on Northern Tenerife and terrestrial deposits from the resulting landslide-tsunamis suggest that initial stages of multistage submarine flank failure were succeeded by the Abrigo caldera-forming eruption^12,37^. Here, we investigate this relationship between volcanic island landslides and large explosive, often caldera-forming, Plinian eruptions. We wish to understand whether landslides and eruptions are linked, and if so, which comes first. Past work has shown that large-volume volcanic island collapse can occur in multiple distinct stages or sub-units^12,38^. Therefore we also wish to understand whether particular stages of failure are associated with the eruptions and test previous hypotheses regarding the influence of ocean island flank collapses towards changing magma chamber pressure regimes at depth^39^.

Our study examines distal marine deposits, called turbidites, from the multistage Icod, Orotava and Roques de Garcia flank collapses on Northern Tenerife. The ages of these deposits coincide with the timing of Abrigo and Granadilla eruptions that generated the Diego Hernandez and Guajara calderas, respectively, and ignimbrite eruptions that created the Ucanca caldera (Fig. 2; Supplementary 1)^7,12,27,29–31,40–44^. We compare chemical compositions of volcanic glass from multiple turbidite layers (sub-units) of these individual slides deposited in the basins adjacent to the Western Canary Islands. These data represent newly analysed volcanic glasses and those collated from past studies^12,38^. We also analyse volcanic glasses from the coeval Abrigo and Granadilla eruptions recovered from terrestrial exposures near the town of Tajao, Southern Tenerife. We also collate previous analyses of volcanic glasses from a tephra recovered in ODP Core 953 dated to ~1.0 Ma synonymous with the caldera-eruptions at the end of the Ucanca Formation^45,46^. Comparison of compositions of volcanic glasses from the landslides and eruptions provides a uniquely detailed investigation of the landslide dynamics and their relationships with island volcanism (Supplementary 1, 2)^6,7,38^.

### Tenerife’s flank collapses and their relationship to caldera eruptions

The Las Cañadas volcanic edifice on Tenerife has a history punctuated by both effusive lava flows and explosive eruptions of phonolitic ignimbrites^31,47^. Indeed, recent volcanism has occurred within three cycles, each culminating in a caldera-forming eruption, these include the most recent Abrigo (160–185 ka) at the end of the Diego Hernandez Cycle, Granadilla (560–600 ka) ignimbrites at the end of the Guajara Cycle, and a series of ignimbrites (1.0–1.2 Ma) at the end of the Ucanca Cycle (Supplementary 1–2)^31,40–42,47^. The northern flank of Tenerife is sculpted by the voluminous multistage Icod (165 ± 5 ka), Orotava (535 ± 5 ka) and Roques de Garcia (1.15 ± 0.01 Ma) landslides, which broadly coincide with the age of the Abrigo, Granadilla and terminal Ucanca caldera-forming eruptions (Fig. 2; Supplementary 1)^6,7,12,39^, suggesting that the collapses and eruptions are likely linked^7,12,29,30,37^; although the nature of that link remained unresolved with certainty.

**Figure 3:**
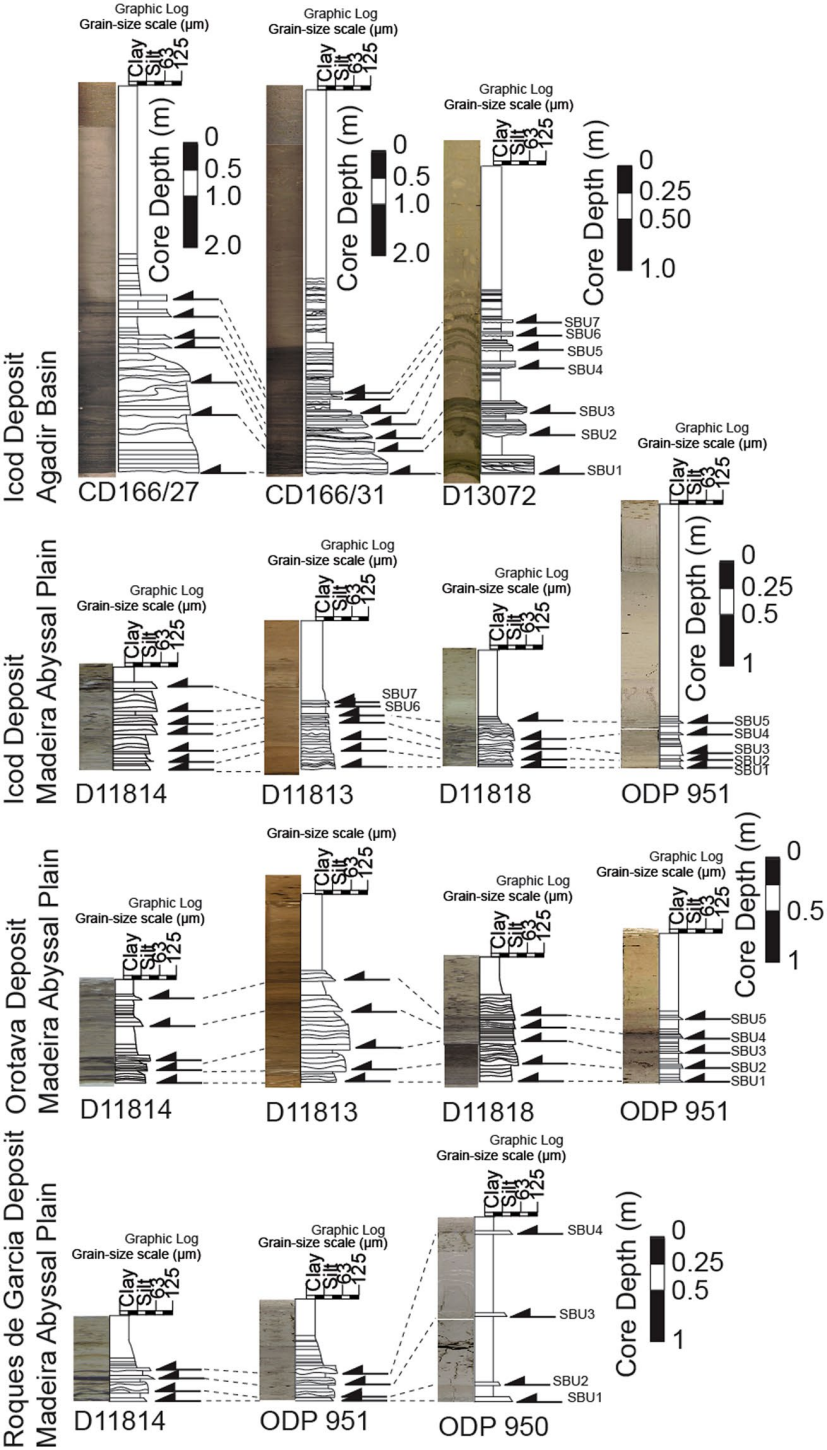
Deposits from the Icod, Orotava and Roques de Garcia landslides from Tenerife recovered from the Agadir Basin and Madeira Abyssal Plain. Deposits show the presence of subunits, with multiple fining-upwards sands that are amalgamated proximally but form discrete sand-mud couplets distally. Distally from source these individual turbidite sub-units are separated by 5–75 cm of turbidite mud representing fine suspension fallout that has settled and consolidated between the flows of each failure stage. SBU stands for sub-unit.

Turbidites were deposited beneath dilute turbulent sediment flows formed as the slide mass moved downslope under gravity and disaggregated^7,8,12,38^. Canary Island landslide deposits in adjacent deep sea basins, such as those of the Icod, Oratava and Roques de Garcia landslides, are unusual as they comprise a series of stacked graded turbidite sand and mud intervals, called sub-units (Fig. 3)^12,38,48,49^. These sub-units are compositionally distinct and record landslide emplacement into the ocean in a series of retrogressive stages^12,38,49^. In these distal deep-sea environments over 500 km from the islands, each turbidite sub-unit is often separated by a 5–75 cm layer of clay and fine silt. These fine-grained intervening sediments settled relatively slowly from the waning turbidity current, potentially reflecting substantial time lags (representing at least tens to hundreds of hours) between individual successive sub-unit failures (Fig. 3)^12^. The initial sub-unit(s) within the distal landslide deposits are thicker and contain marine bioclastic sediment and altered volcaniclastics in contrast to later sub-units^12,38,49^. This suggests that the initial stage(s) of each landslide comprised most of the failure volume, and were sourced from submerged parts of the volcanic island^12,38^. Later sub-units are smaller-volume and are composed almost entirely of unaltered volcanic glass from the flanks of the active subaerial edifice^12,38,49^, implicating progressive sub-unit failures migrated retrogressively from the submerged to the entirely subaerial flanks of the volcano^12,38,49^.

**Figure 4:**
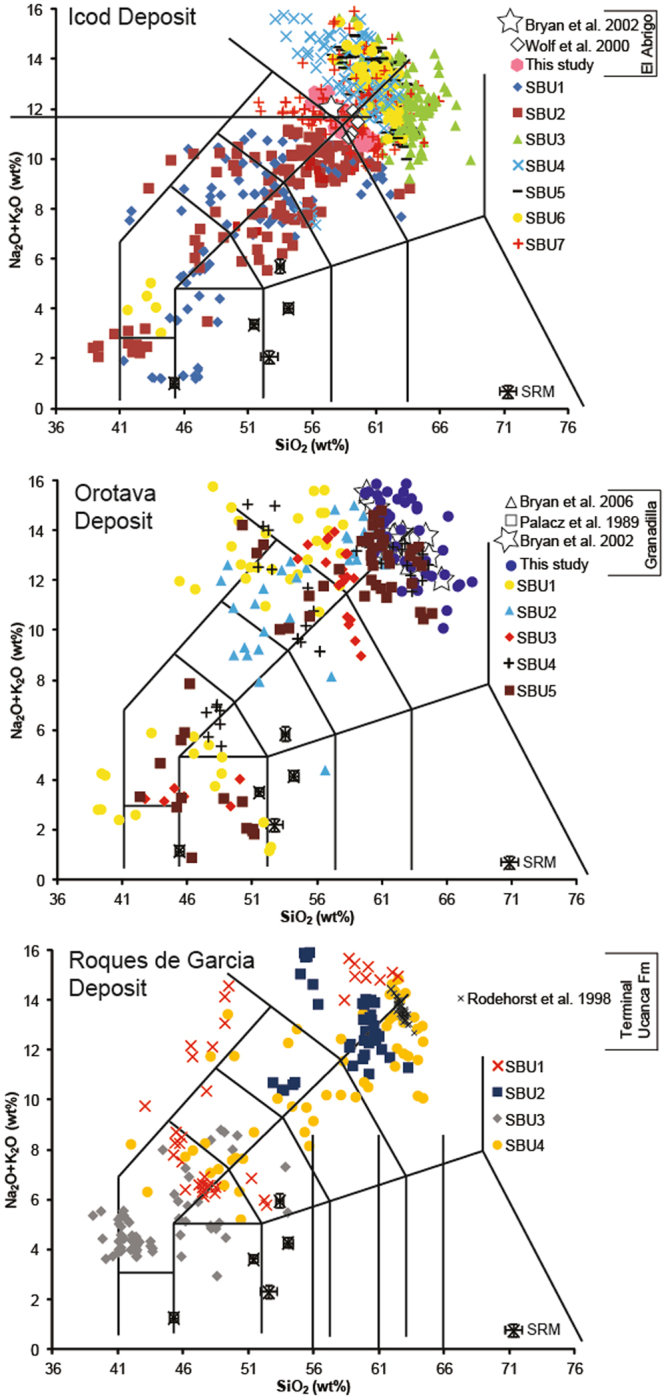
Compositions of volcanic glasses recovered from sub-units of the Icod, Orotava and Roques de Garcia deposits from Tenerife plotted on Total Alkali-Silica (TAS) diagrams. The Icod deposit represents a collation of data from sub-units from core sites from Agadir Basin and the Madeira Abyssal Plain (data from Hunt *et al*.^7,12,49^ and Supplementary 2 inc. 283 new analyses from core D11813 in the Madeira Abyssal Plain), while Orotava and Roques de Garcia subunits are only sampled in the Madeira Abyssal Plain (data from Hunt *et al*.^38^ and Supplementary 2 inc. 150 new analyses from core D11813 and 42 new analyses from D11814 in the Madeira Abyssal Plain respectively). Compositions of Abrigo, Granadilla, and Ucanca ignimbrite glasses and whole rock samples were collated from published work (tephra deposit 1.00–1.12 Ma in ODP 953, Rodehurst *et al*. 1998) and new glasses analyses of the Abrigo (41 new analyses) and Granadilla (47 new analyses) ignimbrites from this study are also plotted for comparison (data from Supplementary 3). Additional composition data of ignimbrite glasses collated from literature^44,45,52,66–68^. Figure show that volcanic glasses explosive volcanic eruptions, including caldera collapse, proposed as being coeval with landslide are only present in the later sub-units, and not present in the initial submarine stages. SBU stands for sub-unit.

We compared the major element composition of volcanic glasses in the sub-units of the Icod and Oratava landslide-turbidites with the volcanic glass compositions of terrestrial ignimbrite deposits of the Abrigo and Granadilla caldera-forming eruptions. These two caldera-related ignimbrites have distinct glass chemical compositions (Fig. 4). Our work confirmed that only the uppermost sub-unit of the Icod deposit (sub-unit 7), the last stage of multi-stage flank collapse, contains volcanic glasses of the same composition as the Abrigo ignimbrite (Fig. 4). A similar relationship is seen for the Orotava deposit, where only the uppermost sub-units (sub-units 4 and 5) contain volcanic glasses that are consistent with the Granadilla ignimbrite (Fig. 4). Importantly, glasses of compositions representing these ignimbrites are not found in initial sub-units of either Icod or Orotava failure (Fig. 4). This implies that the lattermost stages of landsliding during each multi-stage landslide took place following the deposition of the respective ignimbrites; so the caldera-related eruptions preceded these last subaerial landslide stages in a temporal sequence, but succeeded an initial series of submarine flank landslides.

The Abrigo and Granadilla eruptions had volumes of 10 and 20 km^3^, respectively^28^, and likely rapidly covered the subaerial part of the island with erupted material within a matter of hours. Tephra fallout from the eruption columns and co-genetic pyroclastic density currents would emplace erupted material across the island’s submerged slopes most likely within hours to days from the start of the eruption^50,51^. Thus should the eruptions have occurred prior to commencement of flank collapse, there should have been both the volume and spatial distribution for that material to be present in the initial stages of the Icod and Orotava flank collapses. Our results imply that the initial failure(s) occurred prior to and thus potentially triggered the final most explosive eruptions in each respective eruption cycle, which in these cases were caldera-forming eruptions. Whilst the landslides do not specifically cause caldera-collapse eruptions, as the magmatic system would have independently developed conditions towards such an eruption, the landslide occurrence may instead escalade the final eruption or induce its occurrence earlier than expected.

A question remains what ultimately triggered the initial submarine stages of each multi-stage landslide leading to the subsequent caldera-collapse eruption and further subsequent subaerial mass wasting? Indeed, could earlier pre-eruptive seismic activity or smaller escalating eruptions trigger the multi-stage flank collapse and commence the aforementioned scenario? Each of the Abrigo and Granadilla terrestrial volcanic successions show a series of pumaceous lapilli fall deposits before the final ignimbrites and tephras representing the caldera collapse eruptions^28,42,52^. Thus eruptive activity prior to and escalating towards the final caldera-forming eruption may be responsible for triggering the initial submarine stages of flank collapse and inducing the aforementioned scenario.

The Roques de Garcia landslide from Northern Tenerife occurred at 1.15 ± 0.01 Ma, dated from an event bed in the offshore Madeira Abyssal Plain^7,8^, supports evidence from the Icod and Orotava landslides. This slide is also represented in the deep sea by a series of stacked turbidites representing multistage collapse, similar to the Icod and Orotava deposits (Fig. 3)^38^. This flank collapse occurs coincidentally with a tephra recovered in ODP Cores 953, 954 and 956 (~1.00–1.15 Ma) that represents an ignimbrite eruption at the end of the Ucanca Formation (1.05 Ma)^45,46^. Volcanic glasses of terminal Ucanca ignimbrite eruptions have compositions only present in the uppermost subunit (sub-unit 4) (Fig. 4). This supports evidence from the Icod and Orotava landslides, whereby the initial submarine stage(s) of flank collapse likely triggered by volcanic activity or related seismicity are followed by a major explosive eruption, in this case caldera-collapse, and finally the last stage(s) of subaerial flank collapse that contain material from the preceding eruption.

### Discussion of landsliding influence on explosive volcanism

Our study importantly demonstrates that whilst early eruptive activity may trigger initial large-volume submarine stages of flank collapse on Tenerife, these initial submarine stages of flank collapse occurred before caldera collapse and potentially induce the eventual explosive eruption. This is demonstrated by only the last stage(s) of the Icod, Orotava and Roques de Garcia failures containing glasses of the respective coeval eruption, thus occurring post-eruption, whilst initial stages are devoid of volcanic glasses of such compositions and are pre-eruption (Figs 3–4). This data supports models that suggest the significant pressure changes involved with large-scale removal of surface material during flank collapse may be responsible for inducing caldera-collapse eruptions^39,43–45^. This is through means of forced decompression on the magma chamber by removal of overburden by landsliding.

One suggestion is the volcanic island flank collapse leads to decompression of relatively shallow magma chambers likely present during the Ucanca, Granadilla and Abrigo caldera-forming eruptions. Although whilst the landslides induced the final eruptions, the style and explosivity of those eruptions were likely constrained by the properties of the magma chamber that had developed. However, the scenario may be more complicated, at least for the 165 ka phonolitic Abrigo eruption. The Abrigo magma was generated at a pressure of 130 ± 50 MPa at a relatively shallow depth of 4–5 km, but was originally water and volatile undersaturated during magma storage^53^. It is suggested that an input of volatile-rich mafic magma from depth was likely needed to trigger the caldera-collapse eruption. Numerical modelling demonstrates that landslide-induced unloading of about 3% of an island mass can cause a pressure reduction of several MPa at shallow depths below ocean islands^39^. This pressure reduction deteriorates exponentially with depth but is still significant at 18–26 km depths beneath Tenerife where primitive basaltic magmas likely accumulate and differentiate^39,53–56^. The landslide decompression may have had the effect of instigating the basaltic magma to rise. Despite landslide-induced decompression the shallow Abrigo magma was water and volatile undersaturated (~3 wt% H_2_O), and may not have erupted explosively^53,55^. Mixing with the deeper ascending volatile-rich basaltic magma may have instigated such an explosive eruption represented by the Abrigo caldera-collapse^53–56^. Effusion of less evolved lavas with high volatile contents follow large flank collapses and ignimbrite eruptions on Tenerife, supporting the notion of deep magma ascent following induced decompression^29,39,53,54,57^.

For the Icod, Orotava and Roques de Garcia landslides on Tenerife we suggest the following scenario. Firstly, early volcanic activity may initially trigger lateral volcanic island collapse of the submarine flank. Static depressurisation effect caused resulting from flank collapse decompress a shallow magma chamber and induces eruption, and in these cases caldera-forming eruptions. However, where magma compositions were too volatile-undersaturated for such eruptions to commence, static decompression may have caused liberation of mafic, volatile-rich magmas at great depths that ascended and mixed with the shallower volatile under-saturated magma, and finally triggered an explosive eruption^29,39,54^. The static decompression affects of landslides on ocean island magmatic systems is not restricted to the Canary Islands, with similar effects evidenced from multistage collapses on Montserrat, in the Lesser Antilles^57,58^.

### Multistage collapse, explosive eruptions and tsunamis

**Figure 5:**
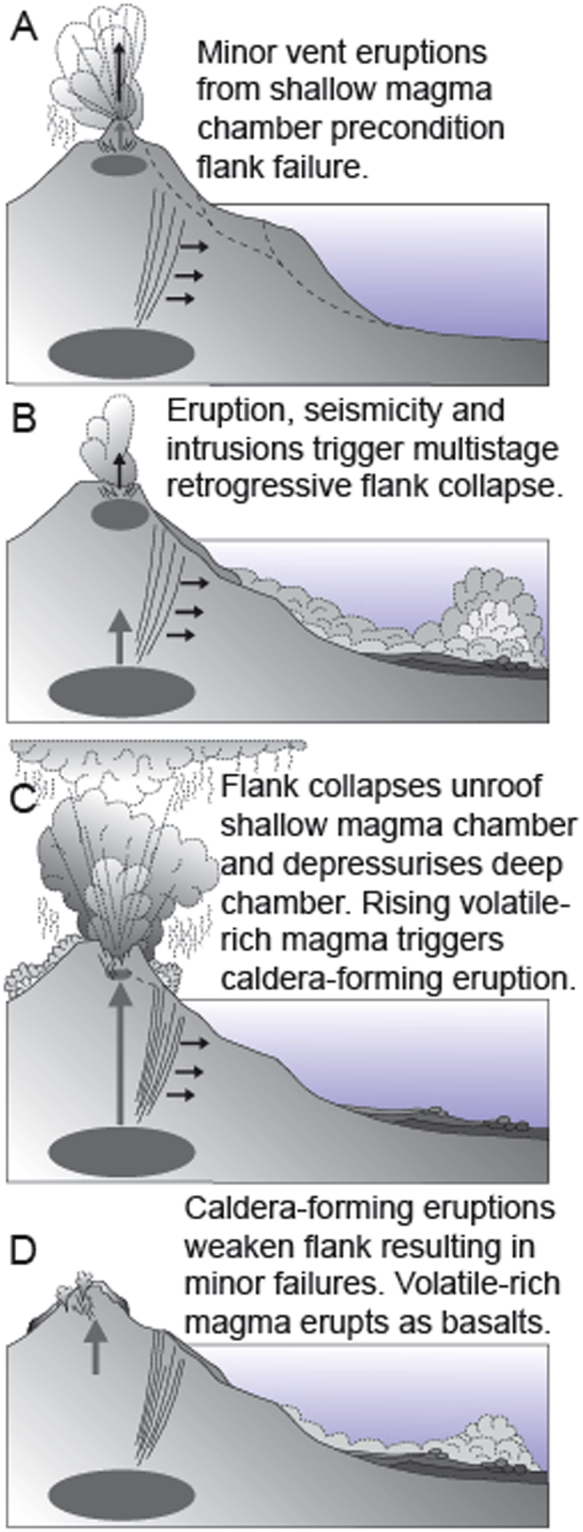
Idealised model for the relationship between the multistage Icod, Orotava and Roques de Garcia landslides and the Abrigo, Granadilla and terminal Ucanca Formation caldera-forming eruptions. (**A**) Minor vent related volcanism and shallow magma chamber activity precondition the submarine slopes to fail. (**B**) Minor eruptions and related seismicity trigger initial submarine stages of multi-stage landslides. (**C**) Submarine landslides unroof both shallow and deep magma chambers, this either (i) caused static decompression in the shallow magma chamber causing immediate explosive eruption, or (ii) static decompression at depth causing ascension of volatile-rich basalt magma that mixes with otherwise volatile undersaturated shallow magma to trigger explosive eruption. (**D**) Explosive or caldera-formning eruptions weaken edifice flanks resulting in subaerial stages of multi-stage failure, containing volcanic debris from explosive eruptions.

Here, we present evidence that triggering eruptions by multistage flank failure is not always instantaneous as observed at the lateral blast of the 1980 Mount St. Helens eruption (Fig. 5). Analysis of stacked turbidites of these landslides recovered in sediment cores from the Madeira Abyssal Plain identified between 5 and 75 cm of 2–4 μm clay is deposited between individual sub-units. It has been suggested that to first deposit and then consolidate these fine-grained intervening sediments to resist erosion by successive sub-unit flows would require time lags on an order of hundreds of hours^12^. These time lags have a geodynamic and associated geohazard implication, whereby several days to weeks may pass between successive stages of flank collapse and that similar prolonged time may pass before a triggered explosive caldera eruption may occur (Fig. 5).

Volcanic island flank collapses can also produce destructive tsunamis^7,13,37,56,57,59^. This and other Canary Island flank collapses were multistage, dividing the landslide mass among numerous staggered failures, resulting in reduced tsunami wave amplitudes compared to equivalent single-block subaerial failures, because the volume is the primary control on tsunami amplitude^12,13,38,58^. Modelling of landslide-tsunamis suggest that time lags of at least greater than 20–50 seconds between successive stages of collapse removes effects of wave inference that would otherwise cause increased tsunami wave amplitudes^12,13,60–64^. Our study suggests that time lags on the order of days likely exist between the individual stages of last three significant failures from the northern flank of Tenerife, and as a result the tsunami waves were likely discrete without interference and thus had lower comparative amplitudes^60–64^.

## Methods and Data

The sediment piston cores of this study form a component of previous published research and an established basin stratigraphy^7,8,65^. Examples of the biostratigraphy and hemipelagite lithostratigraphy of these are included in supplement 1. Coccolith biostratigraphy provided a series of datum horizons^7^. Species were counted using a *Hitachi TM1000* SEM at the British Ocean Sediment Core Research Facility (BOSCORF) and the methodology of Hunt *et al*.^7^. Counts were completed at five centimetre intervals and above and below turbidites or changes in hemipelagite lithology.

Volcanic glasses from the individual sub-units of the event beds was completed using the *SwiftEd* EDS as part of the *Hitachi TM1000 SEM* at BOSCORF and the methodology of Hunt *et al*.^38^ (Supplementary 2). Samples from each sub-unit were sieved at 63 μm and subjected to acetic acid (0.1 M) leaching to remove carbonate. For the Icod slide sub-units were sampled from core D11813 and included 283 new analyses across all sub-units. The Orotava slide sub-units were sampled from core D18813 with 150 new analyses, the Roques de Garcia slide sub-units were sampled from core D11814 with 42 new analyses, and the Cumbre Nueva slide sub-units were sampled from core D11818 with 28 new analyses. Grains were mounted on semi-conductor pads and 30–50 volcanic glasses 90–125 μm and >45 μm thick were analysed for 120 s at 15 kV. Samples were recovered from the El Abrigo and Granadilla ignimbrites on Tenerife and analysed using the *SwiftEd* EDS as part of the *Hitachi TM1000 SEM* at BOSCORF and the methodology of Hunt *et al*.^38^ (Supplementary 3). These data were validated for accuracy and precision using a series of international standard reference materials (SRMs) as analytical glasses, produced using methodology of Hunt *et al*.^38^ (Supplementary 4). Concentrations between 1 and 2 wt% had precisions of 5.0–10.2% of the value, concentrations between 2 and 10 wt% had precisions of 1.0–7.5%, while those values >10 wt% had precisions within <1.5% of the value. Accuracies of SEM EDS results compared to certificated data were generally within 0.3–10.0% of the SRM certified value for values >1 wt%. Specifically, SiO_2_ that ranged from 45.35 to 53.41 wt% among SRMs had accuracies to within 0.3–3.7% of the certified value. For Na_2_O that ranged from 1.13 to 3.64 wt% among SRMs had accuracies to within 5.8% of the certified value. Values of K_2_O among the SRMs only ranged from 0.25 to 2.60 wt%, including three SRMs <0.35 wt%, and had precisions on average within 7.2% of the certified value. Generally, values of Na_2_O and K_2_O within volcanic glasses analysed were >2.5 wt% and >4.0 wt%, respectively and thus yielded both improved precision and accuracy.

### Data availability statement

All geochemical data pertaining to the compositions of volcanic glasses from each of the landslide sub-units and their associated caldera-forming eruptions is supplied within the supplementary data. Any addition data can be made available upon request of the lead author.

## Electronic supplementary material

Supplementary Information

## References

[1] Duffield WA, Stieljes L, Varet J (1982). Huge landslide blocks in the growth of Piton de la Fournaise, La Reunion, and Kilauea volcano, Hawaii. J. Volcanol. Geotherm. Res..

[2] Moore JG (1989). Prodigious submarine landslides on the Hawaiian Ridge. J. Geophys. Res..

[3] Moore JG, Normark WR, Holcomb RT (1994). Giant Hawaiian landslides. Ann. Rev. Earth Planet. Sci..

[4] Masson DG (2002). Slope failures on the flanks of the western Canary Islands. Earth Sci. Rev..

[5] Acosta J (2003). Geologic evolution of the Canary Islands of Lanzarote, Fuerteventura, Gran Canaria and La Gomer and comparison of the landslides at these islands with those at Tenerife, La Palma and El Hierro. Mar. Geophys. Res..

[6] Masson, D. G., Le Bas, T., Grevemeyer, I. & Weinrebe, W. Flank collapse and large-scale landsliding in the Cape Verde Islands, off West African. *Geochem. Geophys. Geosy*. **9** (2008).

[7] Hunt JE, Wynn RB, Talling PJ, Masson DG (2013). Turbidite record of frequency and source of large volume (>100 km^3^) Canary Island landslides in the last 1.5 Ma: implications for landslide triggers and geohazards. Geochem. Geophys. Geosys..

[8] Hunt JE, Talling PJ, Clare MA, Jarvis I (2014). Long-term (17 Ma) turbidite record of the timing and frequency of large flank collapses of the Canary Islands. Geochem. Geophys. Geosys..

[9] Watt S, Talling PJ, Hunt JE (2014). New insights into the emplacement dynamics of volcanic island landslides. Oceanography.

[10] Tappin D (2001). The Sissano, Papua New Guinea tsunami of July 1998 – offshore evidence of the source mechanism. Mar. Geol..

[11] Ward SN, Day SJ (2001). Cumbre Vieja volcano: potential collapse and tsunami at La Palma, Canary Islands. Geophys. Res. Lett..

[12] Hunt JE (2011). Sedimentological and geochemical evidence for multistage failure of volcanic island landslides: A case study from Icod landslide on north Tenerife, Canary Islands. Geochem. Geophys. Geosys..

[13] Giachetti T (2011). Numerical modelling of the tsunami triggered by the Guimar debris avalanche, Tenerife (Canary Islands): comparison with field-based data. Mar. Geol..

[14] Paris R (2015). Source mechanisms of volcanic tsunamis. Phil. Trans. Royal Soc..

[15] Waitt RB, Hansen VL, Wood SH (1981). Devastating pyroclastic density flow and attendant air fall of May 18 – Stratigraphy and sedimentology deposits. U.S. Geol. Survey Prof. Paper.

[16] McEwen AS, Malin MS (1989). Dynamics of Mount St. Helens’ 1980 pyroclastic flows, rockslide-avalanche, lahars, and blast. J. Volcanol. Geotherm. Res..

[17] Malone, S.D. *et al*. In *The 1980 Eruption of Mount St. Helens, Washington* (eds Lipman, P. W., & Mullineaux, D. R.) 808–813 (U. S. Geol. Surv. Prof. Pap. 1250, 1981).

[18] Hoblitt RP (2000). Was the 18 May 1980 lateral blast at Mt St Helens the product of two explosions?. Philosophical Transactions of the Royal Society of London A: Mathematical, Physical and Engineering Sciences.

[19] Baxter PJ (1981). Mount St Helens eruptions, May 18 to June 12, 1980: an overview of the acute health impact. Jama.

[20] Belousov A (1996). Deposits of the 30 March 1956 directed blast at Bezymianny volcano, Kamchatka, Russia. Bulletin of Volcanology.

[21] Belousov A, Belousova M, Voight B (1999). Multiple edifice failures, debris avalanches and associated eruptions in the Holocene history of Shiveluch volcano, Kamchatka, Russia. Bulletin of Volcanology.

[22] Ponomareva, V. V., Pevzner, M. M. & Melekestev, I. V. Large debris avalanches and associated eruptions in the Holocene eruptive history of Shiveluch volcano, Kamchatka, Russia. *Bulletin of Volcanology* **59**(7), 490–505 (1998).

[23] Ponomareva, V. V., Melekestev, I. V. & Dirksen, O. V. Sector collapses and large landslides on Late Pleistocene-Holocene volcanoes in Kamchatka, Russia. *J. Volcanol. Geotherm. Res*. **158**(1), 117–138 (2006).

[24] Siebert L, Glicken H, Ui T (1987). Volcanic hazards from Bezymianny-and Bandai-type eruptions. Bulletin of Volcanology.

[25] Belousov A, Voight B, Belousova M (2007). Directed blasts and blast-generated pyroclastic density currents: a comparison of the Bezymianny 1956, Mount St Helens 1980, and Soufrière Hills, Montserrat 1997 eruptions and deposits. Bulletin of Volcanology.

[26] Carey S, Sigurdsson H, Gardner JE, Criswell W (1990). Variations in column height and magma discharge during the May 18, 1980 eruption of Mount St. Helens. J. Volcanol. Geotherm. Res..

[27] Huertas MJ (2002). 40Ar/39Ar stratigraphy of pyroclastic units from the Canadas Volcanic Edifice (Tenerife, Canary Islands) and their bearing on the structural evolution. J. Volcanol. Geotherm. Res..

[28] Edgar CJ (2007). The late Quaternary Diego Hernandez Formation, Tenerife: Volcanology of a complex cycle of voluminous explosive phonolitic eruptions. J. Volcanol. Geotherm. Res..

[29] Martí J, Hurlimann M, Ablay GJ, Gudmundsson A (1997). Vertical and lateral collapses on Tenerife (Canary Islands) and other volcanic ocean islands. Geology.

[30] Hurlimann M, Ledesma M, Marti J (1999). Conditions favouring catastrophic landslides on Tenerife (Canary Islands). Terra Nova.

[31] Hurlimann M, Turon E, Marti J (1999). Large landslides triggered by caldera collapse events in Tenerife, Canary Islands. Phys. Chem. Earth. Part A.

[32] Hürlimann M, Martí J, Ledesma A (2000). Mechanical relationship between catastrophic volcanic landslides and caldera collapses. Geophys. Res. Letts..

[33] Boulesteix T, Hildenbrand A, Gillot PY, Soler V (2012). Eruptive response of oceanic islands to giant landslides: new insights from the geomorphic evolution of the Teide-Pico Veijo volcanic complex (Tenerife, Canary). Geomorphology.

[34] Boulesteix T, Hildenbrand A, Soler V, Quidelleur X, Gillot PY (2013). Coeval giant landslides in the Canary Islands: implications for global, regional and local triggers of giant flank collapses on oceanic volcanoes. J. Volcanol. Geotherm. Res..

[35] Martí J, Mitjavila J, Araña V (1994). Stratigraphy, structure and geochronology of the Las Cañadas caldera (Tenerife, Canary Islands). Geological Magazine.

[36] Tomassi, P., *et al*. In *Landslides* (eds Sassa, K., Fukuoka, H., Wang, F., & Wang, G.) 251–258 (Springer, 2005).

[37] Paris, R., Bravo, J. J. C., González, M. E. M., Kelfoun, K., & Nauret, F. Explosive eruption, flank collapse and megatsunami at Tenerife ca. 170 ka. *Nature Communications*, **8** (2017). 10.1038/ncomms15246 PMC5440666 28504256

[38] Hunt JE, Wynn RB, Talling PJ, Masson DG (2013). Multistage collapse of eight western Canary Island landslides in the last 1.5 Ma: Sedimentological and geochemical evidence from subunits in submarine flow deposits. Geochem. Geophys. Geosys..

[39] Manconi A (2009). The effects of flank collapses on volcano plumbing systems. Geology.

[40] Bryan SE, Cas RA, Marti J (1998). Lithic breccias in intermediate volume phonolitic ignimbrites, Tenerife (Canary Islands): constraints on pyroclastic flow depositional processes. J. Volcano. Geotherm. Res..

[41] Brown (2003). The Quaternary pyroclastic succession of southeast Tenerife, Canary Islands: explosive eruptions, relates caldera subsidence, and sector collapse. Geol. Mag..

[42] Pittari A (2006). The influence of palaeotopography on facies architecture and pyroclastic flow processes of a lithic-rich ignimbrite in a high gradient setting: The Abrigo Igrimbrite, Tenerife, Canary Islands. J. Volcanol. Geotherm. Res..

[43] Marti J (2010). Resolving problems with the origin of Las Canadas caldera (Tenerife, Canary Islands): Los Roques de Garcia Formation – Part of a major debris avalanche or an *in-situ*, stratified, edifice-building succession?. Geol. Soc. Am. Spec. Pub..

[44] Davila-Harris P (2013). Lithostratigraphic analysis and geochemistry of a vitric spatter-bearing ignimbrite: the Quaternary Adeje Formatio, Canadas volcano, Tenerife. Bullet. Volcanol..

[45] van den Bogaard, P. In *Proceedings of the Ocean Drilling Programme* (eds Weaver, P. P. E., Schmincke, H. -U., Firth, J. V. & Duffield, W.) 315–328 (Ocean Drilling Programme, 1998).

[46] Rodehorst, U. *et al*. In *Proceedings of the Ocean Drilling Programme* (eds Weaver, P. P. E., Schmincke, H. -U., Firth, J. V. & Duffield, W.) 315–328 (Ocean Drilling Programme, 1998).

[47] Bryan SE, Marti J, Leosson M (2002). Petrology and geochemistry of the Bandas del Sur Formation, Las Canadas edifice, Tenerife (Canary Islands). J. Petrol..

[48] Wynn, R. B., & Masson, D. G. In *Submarine mass movements and their consequences* (eds Locat, L. & Mienert, J.) 325–332 (Springer Netherlands, 2003).

[49] Hunt, J. E., Wynn, R. B., & Croudace, I. W. In *Micro-XRF Studies of Sediment**Cores*, (eds Croudace, I. W. & Rothwell, R. G.) 147–172 (Springer Netherlands, 2015).

[50] Trofimovs J (2006). Submarine pyroclastic deposits formed at the Soufriere Hills volcano, Montserrat (1995–2003): What happens when pyroclastic flows enter the ocean?. Geology.

[51] Guillou H, Carracedo JC, Day SJ (1998). Dating of the upper Pleistocene–Holocene volcanic activity of La Palma using the unspiked K–Ar technique. J. Volcanol. Geotherm. Res..

[52] Bryan SE (2006). Petrology and geochemistry of the Quaternary caldera-forming, phonolitic Granadilla eruption, Tenerife (Canary Islands). J. Petrol..

[53] Andujar J (2008). Experimental constraints on pre-eruptive conditions of phonolitic magma from the caldera-forming El Abrigo eruption, Tenerife (Canary Islands). Chem. Geol..

[54] Klügel A, Hansteen TH, Galipp K (2005). Magma storage and underplating beneath Cumbre Vieja volcano, La Palma (Canary Islands). Earth Planet. Sci. Letts..

[55] Andujar J, Scaillet B (2012). Relationships between pre-eruptive conditions and eruptive styles of phonolitic-trachyte magmas. Lithos.

[56] Stroncik NA, Klugel A, Hansteen TH (2009). The magmatic plumbing system beneath El Hierro (Canary Islands): constraints from phenocrysts and naturally quenched basaltic glasses in submarine rocks. Contrib. Mineral. Petrol..

[57] Cassidy M (2014). Multi-stage collapse events in the South Soufriere Hills, Montserrat as recorded in marine sediment cores. Geol. Soc. London. Mem..

[58] Murty TS (2003). Tsunami wave height dependence on landslide volume. Pure and Applied Geophysics.

[59] Cassidy M (2015). Rapid onset of mafic magmatism facilitated by volcanic edifice collapse. Geophys. Res. Letters.

[60] Masson DG (2006). Submarine landslides: processes, triggers and hazard prediction. Philosophical Transactions of the Royal Society of London A: Mathematical, Physical and Engineering Sciences.

[61] Harbitz CB (2006). Mechanisms of tsunami generation by submarine landslides: a short review. Norwegian Journal of Geology/Norsk Geologisk Forening.

[62] Haugen KB, Løvholt F, Harbitz CB (2005). Fundamental mecha- nisms for tsunamigeneration by submarine flows in idealised geometries. Mar. Pet. Geo..

[63] Løvholt F., Pedersen G., Harbitz C. B. Tsunamigenesis due to retrogressive landslides on an inclined seabed. In Submarine mass movements and their consequences (eds Lamarche, G. *et al*.), vol. 7. Berlin, Germany: Springer (2015a).

[64] Løvholt F, Pedersen G, Harbitz CB, Glimsdal S, Kim J (2015). On the characteristics of landslide tsunamis. Phil. Trans. R. Soc..

[65] Weaver PPE, Kuijpers A (1983). Climatic control of turbidite deposition on the Madeira Abyssal Plain. Nature.

[66] Palacz ZA, Wolff JA (1989). Strontium, neodymium and lead isotope characteristics of the Granadilla Pumice, Tenerife: a study of the causes of strontium isotope disequilibrium in felsic pyroclastic deposits. Geol. Soc. London Spec. Pub..

[67] Wolff JA, Grandy JS, Larson PB (2000). Interaction of mantle-derived magma with island crust? Trace element and oxygen isotope data from the Diego Hernandez Formation, Las Cañadas, Tenerife. J. Volcanol. Geotherm. Res.

[68] Carey S (1997). Influence of convective sedimentation on the formation of widespread tephra fall layers in the deep sea. Geology.

