# Supplementary material for: Multi-stage volcanic island flank collapses with coeval explosive caldera-forming eruptions
Source: Sci Rep. 2018 Jan 18;8:1146. doi: 10.1038/s41598-018-19285-2 (PMC5773679; doi:10.1038/s41598-018-19285-2)
Supplement: Supplementary file 1 — Supplementary Information [file 41598_2018_19285_MOESM1_ESM.doc]

**Multi-stage volcanic island collapses trigger explosive caldera-forming eruptions**

James E. Hunt^1*^, Michael Cassidy^2^, and Peter J. Talling^3^

^1^National Oceanography Centre, Southampton, European Way, Southampton, SO143ZH, UK

^2^Department of Earth Sciences, University of Oxford, South Parks Road, Oxford, OX13AN, UK

^3^Departments of Earth Sciences and Geography, Durham University, Durham DH1 3LE, UK

^*^corresponding author: [james.hunt@noc.ac.uk](mailto:james.hunt@noc.ac.uk)

**Supplementary Information**

**Supplement 1. Characteristics of the last major northern flank collapses and coeval major caldera-forming eruptions on Tenerife.**

| Eruption | Eruption Date (ka) | Eruption Deposits | Post Eruption Deposits | Associated Landslide | Landslide Date (ka) | Landslide Deposits |
| --- | --- | --- | --- | --- | --- | --- |
| El Abrigo | 168±1 [1,2]  179±11 [3] | Basal unit (Sur-A) comprising two pumice lapilli deposits each capped with a lithic-concentrated zone.[4,5,6]  This basal unit is followed by a welded tuff (Sur-B) and finally a decimetre-thick lithic-rich lapilli containing ignimbrite lobes capped with a tuff and epiclastics above.[4,5,6] | The El Abrigo member is then followed by the Recent Basalts from the Teide-Pico Viejo system.[1,7,8] | Icod | 165±5 [9,10]  150-170 [11]  173-176 [12]  179-183 [13] | Seven stages of failure commencing offshore.[14] |
| Granadilla | 570 [15]  600±7 [16] | Four main fall units: Unit 1 of four phases of pumice fall and local gravity flows, Unit 2 of cemented-ash representing a phreatomagmatic eruption, Units 3 and 4 represented by metre-thick pumaceous fallout. [7,17] These are capped by a tephra and a metre-thick welded ignimbrite.[17] | This eruptive cycle is then followed by the Cycle 3 basaltic lavas.[16,17] | Orotava | 535±10 [10]  540-690 [11]  526±8 to 534±9 [8]  566±7 [18]  560±20 [19]  505-566 [20] | Five stages of failure commencing offshore.[14] |
| Caldera del Rey | 1007±4 [21]  1130±30 [21] | The Caldera del Rey deposits consist of a basal welded lapilli, then a pumice lapilli, followed by the first welded lapilla tuff with accretionary lapilli, a second pumice lapilli, then a massive tuff with accretionary lapilli, a third but thin pumice lapilli follows, and finally a cross-stratified tuff.[22] | Above this sequence and at the base of the Guajara formation is a sequence of layered basalts. [16,17,22] | Roques de Garcia | 1180±20 [10]  600-1700 [23]  >600 [11] | Four stages of failure.[14] |

[1] Bryan et al., 2002; [2] Brown et al., 2003; [3] Marti et al., 1994; [4] Pittari et al., 2005; [5] Pittari et al., 2006; [7] Edgar et al., 2007; [8] Boulesteix et al., 2012; [9] Hunt et al., 2011; [10] Hunt et al., 2013a; [11] Masson et al., 2002; [12] Mitijavila et al., 1990; [13] Mitijavila and Villa, 1993; [14] Hunt et al., 2013b; [15] Bryan et al., 1998; [16] Brown et al., 2003; [17] Bryan et al., 2000; [18] Carracedo et al., 2001; [19] Ancochea et al., 1990; [20] Krastel et al., 2001; [21] Huertas et al., 2002; [22] Davila-Harris et al., 2013; Acosta et al., 2003.

**Supplement 2. SEM EDS major element compositions of volcanic glasses from the individual sub-units from the Icod, Orotava, and Roques de Garcia submarine landslides from Tenerife, western Canary Islands.**

**2.1 Icod volcanic glass compositions in wt%**

| Name | Sub-unit | SiO_2_ | TiO_2_ | Fe_2_O_3_T | Al_2_O_3_ | MnO | MgO | CaO | Na_2_O | K_2_O | P_2_O_5_ | Total |
| --- | --- | --- | --- | --- | --- | --- | --- | --- | --- | --- | --- | --- |
| Spectrum 01 | 1 | 51.50 | 1.02 | 5.22 | 20.67 | 0.07 | 6.67 | 5.27 | 3.50 | 4.18 | 1.89 | 100 |
| Spectrum 02 | 1 | 52.50 | 0.95 | 4.86 | 20.59 | 0.00 | 6.78 | 4.97 | 3.74 | 3.94 | 1.67 | 100 |
| Spectrum 03 | 1 | 52.63 | 0.94 | 5.17 | 20.08 | 0.00 | 7.27 | 4.68 | 4.06 | 3.72 | 1.44 | 100 |
| Spectrum 04 | 1 | 51.84 | 0.23 | 1.94 | 20.84 | 0.00 | 8.83 | 6.99 | 3.15 | 5.55 | 0.64 | 100 |
| Spectrum 05 | 1 | 52.58 | 0.70 | 1.78 | 21.42 | 0.00 | 9.28 | 4.66 | 2.77 | 6.14 | 0.66 | 100 |
| Spectrum 06 | 1 | 54.08 | 0.56 | 3.02 | 18.11 | 0.00 | 10.91 | 3.83 | 2.84 | 5.53 | 1.12 | 100 |
| Spectrum 07 | 1 | 54.08 | 0.64 | 3.64 | 17.84 | 0.35 | 10.16 | 3.76 | 2.83 | 5.47 | 1.23 | 100 |
| Spectrum 08 | 1 | 53.48 | 0.48 | 2.88 | 18.50 | 0.00 | 11.06 | 3.99 | 3.06 | 5.65 | 0.89 | 100 |
| Spectrum 09 | 1 | 51.42 | 1.37 | 7.23 | 19.10 | 0.07 | 7.27 | 1.84 | 5.87 | 3.99 | 1.82 | 100 |
| Spectrum 10 | 1 | 52.77 | 1.54 | 6.06 | 17.78 | 0.29 | 8.14 | 2.42 | 5.40 | 4.45 | 1.14 | 100 |
| Spectrum 11 | 1 | 55.71 | 1.05 | 5.83 | 18.13 | 0.00 | 6.39 | 2.91 | 3.57 | 4.82 | 1.59 | 100 |
| Spectrum 12 | 1 | 54.37 | 0.24 | 2.69 | 20.29 | 0.14 | 8.33 | 4.09 | 3.27 | 5.70 | 0.89 | 100 |
| Spectrum 13 | 1 | 57.09 | 0.96 | 3.92 | 18.47 | 0.21 | 7.50 | 1.07 | 7.21 | 3.34 | 0.23 | 100 |
| Spectrum 14 | 1 | 56.41 | 1.04 | 4.38 | 18.69 | 0.21 | 7.49 | 1.19 | 6.25 | 3.43 | 0.90 | 100 |
| Spectrum 15 | 1 | 56.34 | 1.52 | 4.13 | 18.86 | 0.07 | 6.79 | 1.19 | 7.22 | 3.44 | 0.45 | 100 |
| Spectrum 16 | 1 | 56.60 | 1.28 | 4.39 | 19.07 | 0.36 | 6.29 | 1.14 | 7.13 | 3.41 | 0.34 | 100 |
| Spectrum 17 | 1 | 56.96 | 0.88 | 4.65 | 18.10 | 0.35 | 6.66 | 1.13 | 7.20 | 3.38 | 0.68 | 100 |
| Spectrum 18 | 1 | 52.19 | 1.80 | 6.68 | 17.34 | 1.16 | 8.44 | 1.16 | 7.28 | 3.48 | 0.46 | 100 |
| Spectrum 19 | 1 | 52.15 | 0.89 | 6.47 | 18.65 | 1.08 | 9.82 | 0.89 | 6.24 | 3.24 | 0.57 | 100 |
| Spectrum 20 | 1 | 53.43 | 0.65 | 6.57 | 18.34 | 0.72 | 7.90 | 1.52 | 6.34 | 3.73 | 0.80 | 100 |
| Spectrum 21 | 2 | 52.04 | 1.37 | 6.38 | 18.66 | 0.07 | 7.67 | 3.86 | 3.22 | 5.58 | 1.14 | 100 |
| Spectrum 22 | 2 | 51.90 | 0.96 | 5.07 | 18.66 | 0.28 | 7.82 | 4.22 | 3.68 | 5.85 | 1.58 | 100 |
| Spectrum 23 | 2 | 56.51 | 1.59 | 5.88 | 18.26 | 0.14 | 8.91 | 2.87 | 1.88 | 2.28 | 1.68 | 100 |
| Spectrum 24 | 2 | 52.43 | 1.36 | 7.73 | 17.49 | 0.21 | 9.45 | 3.01 | 4.25 | 2.39 | 1.68 | 100 |
| Spectrum 25 | 2 | 51.61 | 1.11 | 8.14 | 17.33 | 0.42 | 9.11 | 2.94 | 5.31 | 2.34 | 1.68 | 100 |
| Spectrum 26 | 2 | 53.36 | 1.03 | 4.50 | 19.30 | 0.42 | 6.65 | 3.76 | 4.72 | 5.47 | 0.79 | 100 |
| Spectrum 27 | 2 | 53.27 | 0.63 | 1.67 | 24.91 | 0.21 | 11.62 | 0.68 | 3.02 | 2.99 | 1.00 | 100 |
| Spectrum 28 | 2 | 56.82 | 1.35 | 3.66 | 20.09 | 0.56 | 7.79 | 1.62 | 4.00 | 3.77 | 0.34 | 100 |
| Spectrum 29 | 2 | 52.99 | 0.81 | 6.52 | 19.65 | 0.79 | 8.99 | 0.89 | 5.77 | 3.24 | 0.34 | 100 |
| Spectrum 30 | 2 | 53.67 | 0.48 | 6.50 | 18.69 | 0.43 | 8.84 | 0.95 | 6.81 | 3.28 | 0.34 | 100 |
| Spectrum 31 | 2 | 53.79 | 0.97 | 5.94 | 19.12 | 0.64 | 8.29 | 0.70 | 7.03 | 3.06 | 0.45 | 100 |
| Spectrum 32 | 2 | 54.93 | 1.37 | 5.54 | 16.87 | 0.00 | 10.44 | 1.65 | 5.15 | 3.84 | 0.20 | 100 |
| Spectrum 33 | 2 | 54.21 | 1.70 | 5.35 | 18.61 | 0.65 | 10.25 | 1.21 | 4.19 | 3.49 | 0.34 | 100 |
| Spectrum 34 | 2 | 55.13 | 0.97 | 5.65 | 19.36 | 0.07 | 9.82 | 1.21 | 4.07 | 3.48 | 0.23 | 100 |
| Spectrum 35 | 2 | 55.79 | 0.81 | 6.09 | 16.42 | 0.14 | 8.76 | 1.34 | 6.25 | 3.60 | 0.80 | 100 |
| Spectrum 36 | 2 | 56.29 | 1.78 | 6.01 | 17.47 | 0.00 | 8.75 | 1.27 | 4.43 | 3.53 | 0.46 | 100 |
| Spectrum 37 | 2 | 56.18 | 1.27 | 4.23 | 18.56 | 0.14 | 7.49 | 1.38 | 6.72 | 3.58 | 0.45 | 100 |
| Spectrum 38 | 2 | 55.62 | 1.12 | 4.52 | 18.73 | 0.00 | 7.50 | 1.26 | 6.86 | 3.49 | 0.90 | 100 |
| Spectrum 39 | 2 | 54.57 | 2.17 | 4.45 | 17.72 | 0.00 | 9.85 | 1.39 | 5.47 | 3.61 | 0.79 | 100 |
| Spectrum 40 | 2 | 58.63 | 0.48 | 3.30 | 18.54 | 0.42 | 6.96 | 0.94 | 6.95 | 3.23 | 0.56 | 100 |
| Spectrum 41 | 2 | 57.77 | 0.56 | 4.17 | 18.40 | 0.21 | 6.43 | 1.56 | 7.06 | 3.72 | 0.11 | 100 |
| Spectrum 42 | 2 | 58.74 | 0.95 | 2.72 | 19.17 | 0.28 | 6.63 | 0.93 | 7.15 | 3.21 | 0.22 | 100 |
| Spectrum 43 | 2 | 55.10 | 1.36 | 4.96 | 18.43 | 0.00 | 9.00 | 1.39 | 5.93 | 3.60 | 0.23 | 100 |
| Spectrum 44 | 2 | 53.84 | 1.37 | 6.50 | 17.72 | 0.00 | 8.54 | 1.84 | 5.63 | 3.99 | 0.57 | 100 |
| Spectrum 45 | 2 | 54.21 | 0.73 | 5.01 | 18.30 | 0.57 | 8.20 | 1.52 | 7.28 | 3.72 | 0.45 | 100 |
| Spectrum 46 | 2 | 56.08 | 1.36 | 4.79 | 18.05 | 0.36 | 6.48 | 1.38 | 7.00 | 3.60 | 0.90 | 100 |
| Spectrum 47 | 2 | 56.77 | 1.50 | 3.06 | 19.15 | 0.00 | 6.93 | 1.74 | 6.44 | 3.85 | 0.56 | 100 |
| Spectrum 48 | 2 | 56.87 | 0.96 | 4.84 | 18.44 | 0.21 | 6.60 | 1.58 | 6.29 | 3.75 | 0.45 | 100 |
| Spectrum 49 | 2 | 54.74 | 1.60 | 5.19 | 18.20 | 0.00 | 8.17 | 1.39 | 6.77 | 3.60 | 0.34 | 100 |
| Spectrum 50 | 2 | 54.78 | 0.72 | 5.86 | 17.75 | 0.78 | 7.65 | 2.02 | 6.07 | 4.12 | 0.23 | 100 |
| Spectrum 51 | 2 | 53.44 | 1.61 | 5.75 | 18.28 | 0.00 | 8.83 | 1.71 | 5.73 | 3.87 | 0.80 | 100 |
| Spectrum 52 | 2 | 53.80 | 1.69 | 5.02 | 18.47 | 0.43 | 9.12 | 1.96 | 5.11 | 4.06 | 0.34 | 100 |
| Spectrum 53 | 2 | 53.06 | 1.62 | 6.90 | 20.34 | 0.14 | 6.24 | 2.73 | 3.47 | 4.70 | 0.80 | 100 |
| Spectrum 54 | 2 | 52.26 | 1.38 | 8.87 | 19.10 | 0.00 | 5.63 | 2.43 | 4.70 | 4.47 | 1.15 | 100 |
| Spectrum 55 | 2 | 56.72 | 1.04 | 4.28 | 17.84 | 0.35 | 6.87 | 2.07 | 6.37 | 4.13 | 0.34 | 100 |
| Spectrum 56 | 2 | 55.96 | 0.95 | 3.20 | 18.21 | 0.00 | 7.85 | 2.30 | 6.33 | 4.30 | 0.89 | 100 |
| Spectrum 57 | 2 | 55.87 | 1.42 | 3.03 | 18.67 | 0.21 | 7.85 | 1.55 | 7.14 | 3.70 | 0.56 | 100 |
| Spectrum 58 | 2 | 56.26 | 1.03 | 3.53 | 18.52 | 0.21 | 7.58 | 2.00 | 6.24 | 4.07 | 0.56 | 100 |
| Spectrum 59 | 2 | 54.38 | 0.88 | 6.08 | 20.18 | 0.00 | 5.98 | 0.88 | 7.85 | 3.21 | 0.57 | 100 |
| Spectrum 60 | 2 | 57.78 | 1.44 | 3.65 | 19.18 | 0.07 | 6.61 | 1.26 | 5.82 | 3.50 | 0.68 | 100 |
| Spectrum 61 | 2 | 56.15 | 0.88 | 4.98 | 20.00 | 0.57 | 6.69 | 0.44 | 6.64 | 2.85 | 0.79 | 100 |
| Spectrum 62 | 2 | 57.41 | 0.40 | 4.67 | 18.61 | 0.21 | 5.87 | 2.40 | 4.76 | 4.41 | 1.25 | 100 |
| Spectrum 63 | 2 | 52.10 | 1.12 | 7.84 | 20.21 | 1.20 | 6.47 | 1.82 | 7.22 | 1.45 | 0.56 | 100 |
| Spectrum 64 | 2 | 55.39 | 0.24 | 6.12 | 19.74 | 0.35 | 5.32 | 1.63 | 6.51 | 3.79 | 0.90 | 100 |
| Spectrum 65 | 2 | 55.40 | 1.71 | 6.66 | 17.99 | 0.00 | 8.72 | 1.15 | 4.22 | 3.46 | 0.69 | 100 |
| Spectrum 66 | 2 | 55.44 | 0.54 | 4.69 | 19.64 | 0.00 | 10.03 | 0.85 | 7.47 | 0.68 | 0.66 | 100 |
| Spectrum 67 | 2 | 54.10 | 1.47 | 6.87 | 16.94 | 0.36 | 7.46 | 1.67 | 6.30 | 3.88 | 0.92 | 100 |
| Spectrum 68 | 2 | 56.22 | 0.92 | 2.60 | 19.16 | 0.07 | 0.87 | 1.27 | 8.18 | 1.00 | 9.72 | 100 |
| Spectrum 69 | 4 | 61.14 | 1.07 | 3.97 | 18.38 | 0.00 | 0.31 | 1.49 | 6.98 | 5.83 | 0.82 | 100 |
| Spectrum 70 | 4 | 58.89 | 1.07 | 3.87 | 20.37 | 0.22 | 0.31 | 0.84 | 8.50 | 5.63 | 0.31 | 100 |
| Spectrum 71 | 4 | 58.24 | 1.09 | 6.88 | 19.08 | 0.07 | 1.05 | 0.99 | 6.10 | 6.19 | 0.31 | 100 |
| Spectrum 72 | 4 | 55.29 | 1.53 | 6.88 | 19.41 | 0.00 | 0.11 | 1.67 | 7.55 | 7.16 | 0.42 | 100 |
| Spectrum 73 | 4 | 50.93 | 1.64 | 13.39 | 16.65 | 1.46 | 0.68 | 1.36 | 3.65 | 10.02 | 0.23 | 100 |
| Spectrum 74 | 4 | 60.41 | 0.81 | 2.75 | 19.97 | 0.65 | 0.81 | 0.90 | 8.08 | 5.23 | 0.40 | 100 |
| Spectrum 75 | 4 | 62.00 | 1.21 | 2.67 | 19.88 | 0.00 | 0.00 | 0.83 | 7.80 | 5.11 | 0.50 | 100 |
| Spectrum 76 | 4 | 61.02 | 0.65 | 2.68 | 20.22 | 0.00 | 1.01 | 1.02 | 7.71 | 5.38 | 0.30 | 100 |
| Spectrum 77 | 4 | 60.78 | 0.65 | 3.42 | 20.17 | 0.29 | 0.00 | 0.96 | 8.42 | 4.92 | 0.40 | 100 |
| Spectrum 78 | 4 | 60.18 | 0.73 | 3.92 | 19.78 | 0.51 | 0.00 | 1.35 | 7.98 | 4.74 | 0.81 | 100 |
| Spectrum 79 | 4 | 61.22 | 0.65 | 3.52 | 19.69 | 0.65 | 0.00 | 0.84 | 7.87 | 5.36 | 0.20 | 100 |
| Spectrum 80 | 4 | 60.51 | 0.81 | 3.51 | 20.38 | 0.22 | 0.00 | 1.15 | 7.73 | 5.09 | 0.61 | 100 |
| Spectrum 81 | 4 | 60.31 | 1.23 | 3.93 | 19.95 | 0.00 | 0.41 | 1.03 | 7.63 | 5.21 | 0.30 | 100 |
| Spectrum 82 | 4 | 65.4 | 1.77 | 3.00 | 17.52 | 0.00 | 0.00 | 2.65 | 3.94 | 5.57 | 0.12 | 100 |
| Spectrum 83 | 4 | 58.28 | 0.83 | 4.47 | 20.05 | 0.59 | 0.10 | 0.91 | 7.85 | 6.30 | 0.62 | 100 |
| Spectrum 84 | 4 |  | 2.98 | 61.07 | 18.01 | 1.17 | 0.33 | 2.62 | 4.80 | 7.82 | 1.20 | 100 |
| Spectrum 85 | 4 | 57.87 | 2.06 | 5.97 | 19.20 | 0.00 | 0.00 | 1.08 | 4.95 | 8.08 | 0.79 | 100 |
| Spectrum 86 | 4 | 57.50 | 0.76 | 6.51 | 19.52 | 1.05 | 0.21 | 1.27 | 5.91 | 6.94 | 0.32 | 100 |
| Spectrum 87 | 4 | 59.41 | 0.81 | 3.23 | 20.73 | 0.00 | 1.22 | 0.83 | 8.08 | 5.49 | 0.20 | 100 |
| Spectrum 88 | 4 | 59.37 | 1.06 | 4.28 | 20.98 | 0.58 | 0.51 | 0.90 | 7.04 | 5.06 | 0.20 | 100 |
| Spectrum 89 | 4 | 60.97 | 1.06 | 3.44 | 20.14 | 0.58 | 1.52 | 0.96 | 6.28 | 4.84 | 0.20 | 100 |
| Spectrum 90 | 4 | 60.04 | 1.23 | 3.24 | 20.34 | 0.36 | 2.04 | 0.71 | 6.43 | 5.52 | 0.10 | 100 |
| Spectrum 91 | 4 | 60.57 | 0.08 | 4.27 | 21.15 | 0.07 | 2.74 | 0.58 | 5.20 | 5.14 | 0.20 | 100 |
| Spectrum 92 | 4 | 59.52 | 0.49 | 2.78 | 20.82 | 1.39 | 0.61 | 0.26 | 7.19 | 6.63 | 0.31 | 100 |
| Spectrum 93 | 4 | 60.32 | 0.75 | 3.86 | 20.26 | 0.15 | 0.41 | 0.78 | 6.15 | 6.90 | 0.41 | 100 |
| Spectrum 94 | 4 | 60.47 | 0.65 | 3.24 | 20.31 | 0.07 | 1.32 | 0.58 | 7.02 | 5.92 | 0.41 | 100 |
| Spectrum 95 | 4 | 60.51 | 0.33 | 3.94 | 21.24 | 0.65 | 0.82 | 0.52 | 6.07 | 5.52 | 0.41 | 100 |
| Spectrum 96 | 4 | 61.28 | 0.82 | 3.96 | 20.19 | 0.66 | 0.82 | 0.78 | 5.85 | 5.34 | 0.31 | 100 |
| Spectrum 97 | 4 | 61.98 | 0.33 | 3.37 | 21.12 | 0.00 |  | 0.32 | 4.98 | 6.19 | 1.72 | 100 |
| Spectrum 98 | 4 | 61.25 | 1.15 | 3.33 | 21.18 | 0.36 | 0.92 | 0.58 | 5.12 | 6.01 | 0.10 | 100 |
| Spectrum 99 | 4 | 60.30 | 0.51 | 3.19 | 19.06 | 0.15 | 0.54 | 0.85 | 8.71 | 6.23 | 0.46 | 100 |
| Spectrum 100 | 4 | 59.73 | 0.94 | 3.84 | 18.34 | 0.23 | 0.11 | 0.85 | 9.83 | 5.79 | 0.35 | 100 |
| Spectrum 101 | 4 | 58.50 | 0.80 | 4.67 | 19.19 | 0.53 | 0.11 | 0.86 | 8.77 | 6.11 | 0.47 | 100 |
| Spectrum 102 | 4 | 59.99 | 0.95 | 4.52 | 17.50 | 0.23 | 0.43 | 0.85 | 9.13 | 5.93 | 0.47 | 100 |
| Spectrum 103 | 4 | 59.42 | 0.73 | 5.47 | 18.11 | 0.08 | 0.22 | 0.85 | 8.77 | 5.89 | 0.47 | 100 |
| Spectrum 104 | 4 | 58.94 | 0.58 | 4.14 | 19.09 | 0.76 | 0.11 | 0.92 | 8.60 | 5.70 | 1.16 | 100 |
| Spectrum 105 | 4 | 55.62 | 0.68 | 8.91 | 17.66 | 0.87 | 0.45 | 1.51 | 6.40 | 7.78 | 0.12 | 100 |
| Spectrum 106 | 4 | 57.18 | 1.28 | 6.74 | 18.06 | 1.03 | 0.67 | 1.43 | 5.33 | 7.79 | 0.48 | 100 |
| Spectrum 107 | 4 | 57.34 | 1.35 | 3.68 | 17.18 | 0.47 | 0.11 | 2.64 | 8.89 | 8.22 | 0.12 | 100 |
| Spectrum 108 | 4 | 56.27 | 1.58 | 7.33 | 18.13 | 0.08 | 0.11 | 1.50 | 5.96 | 7.71 | 1.33 | 100 |
| Spectrum 109 | 4 | 54.82 | 1.51 | 7.97 | 18.48 | 0.08 | 0.34 | 1.57 | 6.39 | 7.87 | 0.97 | 100 |
| Spectrum 110 | 4 | 55.64 | 1.64 | 5.66 | 17.29 | 0.08 | 0.11 | 3.49 | 8.19 | 6.12 | 1.78 | 100 |
| Spectrum 111 | 4 | 56.63 | 0.91 | 4.62 | 18.18 | 0.47 | 0.67 | 4.09 | 6.50 | 7.57 | 0.36 | 100 |
| Spectrum 112 | 4 | 57.38 | 0.07 | 5.13 | 18.11 | 1.32 | 0.55 | 1.41 | 7.41 | 7.55 | 1.07 | 100 |
| Spectrum 113 | 4 | 55.54 | 1.08 | 4.56 | 21.12 | 0.23 | 0.75 | 0.52 | 9.80 | 5.83 | 0.58 | 100 |
| Spectrum 114 | 4 | 53.08 | 1.60 | 8.71 | 18.48 | 0.16 | 0.11 | 1.38 | 7.08 | 8.54 | 0.85 | 100 |
| Spectrum 115 | 4 | 53.74 | 1.52 | 8.68 | 17.66 | 0.56 | 0.11 | 1.71 | 7.84 | 7.94 | 0.24 | 100 |
| Spectrum 116 | 4 | 60.66 | 0.22 | 4.00 | 19.11 | 0.08 | 0.11 | 1.31 | 8.11 | 6.19 | 0.23 | 100 |
| Spectrum 117 | 4 | 60.23 | 1.01 | 3.48 | 18.92 | 0.75 | 0.11 | 0.59 | 8.95 | 5.74 | 0.23 | 100 |
| Spectrum 118 | 4 | 59.87 | 0.79 | 3.76 | 19.26 | 0.08 | 0.21 | 0.65 | 9.19 | 5.84 | 0.35 | 100 |
| Spectrum 119 | 4 | 59.88 | 0.07 | 4.26 | 18.96 | 0.53 | 0.11 | 0.72 | 9.41 | 5.50 | 0.58 | 100 |
| Spectrum 120 | 4 | 59.87 | 0.85 | 5.14 | 18.84 | 0.90 | 1.05 | 1.47 | 4.64 | 6.93 | 0.32 | 100 |
| Spectrum 121 | 4 | 59.35 | 1.18 | 5.75 | 19.35 | 0.00 | 0.52 | 1.26 | 5.61 | 6.67 | 0.31 | 100 |
| Spectrum 122 | 4 | 56.46 | 0.95 | 5.18 | 18.70 | 0.61 | 0.86 | 0.79 | 10.39 | 5.83 | 0.23 | 100 |
| Spectrum 123 | 4 | 60.17 | 1.24 | 3.99 | 19.71 | 0.44 | 0.83 | 1.17 | 6.14 | 6.00 | 0.31 | 100 |
| Spectrum 124 | 4 | 53.46 | 1.83 | 8.37 | 19.22 | 0.62 | 0.00 | 1.71 | 6.32 | 8.37 | 0.11 | 100 |
| Spectrum 125 | 6 | 60.49 | 0.73 | 2.94 | 20.85 | 0.57 | 0.81 | 0.51 | 7.55 | 5.05 | 0.50 | 100 |
| Spectrum 126 | 6 | 61.08 | 0.73 | 2.61 | 19.86 | 0.29 | 0.30 | 0.96 | 7.96 | 5.59 | 0.61 | 100 |
| Spectrum 127 | 6 | 60.33 | 0.90 | 3.10 | 20.04 | 0.14 | 0.61 | 0.96 | 7.74 | 5.56 | 0.61 | 100 |
| Spectrum 128 | 6 | 60.73 | 0.74 | 3.18 | 19.98 | 0.44 | 0.71 | 1.29 | 6.80 | 5.42 | 0.71 | 100 |
| Spectrum 129 | 6 | 60.56 | 1.46 | 2.95 | 20.08 | 0.00 | 0.51 | 0.96 | 7.35 | 5.13 | 1.01 | 100 |
| Spectrum 130 | 6 | 61.17 | 0.74 | 3.10 | 20.67 | 0.36 | 0.81 | 0.84 | 6.29 | 5.51 | 0.51 | 100 |
| Spectrum 131 | 6 | 59.51 | 1.71 | 5.69 | 17.82 | 0.00 | 1.49 | 2.02 | 4.30 | 7.36 | 0.11 | 100 |
| Spectrum 132 | 6 | 60.03 | 0.49 | 3.45 | 20.30 | 0.00 | 0.00 | 1.09 | 8.47 | 5.66 | 0.51 | 100 |
| Spectrum 133 | 6 | 61.73 | 0.65 | 3.57 | 19.71 | 0.36 | 0.71 | 0.77 | 7.23 | 4.87 | 0.40 | 100 |
| Spectrum 134 | 6 | 61.17 | 0.65 | 2.04 | 20.93 | 0.00 | 0.60 | 0.95 | 8.01 | 5.34 | 0.30 | 100 |
| Spectrum 135 | 6 | 60.48 | 1.06 | 3.16 | 20.21 | 0.29 | 0.91 | 0.45 | 7.71 | 5.33 | 0.40 | 100 |
| Spectrum 136 | 6 | 62.99 | 0.57 | 2.17 | 20.49 | 0.00 | 0.30 | 0.76 | 7.08 | 5.26 | 0.38 | 100 |
| Spectrum 137 | 6 | 62.29 | 0.49 | 2.68 | 20.97 | 0.00 | 0.91 | 0.96 | 5.91 | 5.59 | 0.20 | 100 |
| Spectrum 138 | 6 | 61.87 | 0.49 | 3.16 | 20.47 | 0.00 | 1.32 | 0.96 | 6.15 | 5.18 | 0.40 | 100 |
| Spectrum 139 | 6 | 62.83 | 0.89 | 2.18 | 20.56 | 0.00 | 0.80 | 0.57 | 6.46 | 4.99 | 0.70 | 100 |
| Spectrum 140 | 6 | 60.64 | 0.90 | 3.80 | 20.71 | 0.00 | 0.51 | 0.19 | 7.27 | 5.98 | 0.00 | 100 |
| Spectrum 141 | 6 | 59.30 | 0.90 | 3.39 | 20.37 | 0.22 | 0.20 | 1.16 | 7.65 | 5.89 | 0.92 | 100 |
| Spectrum 142 | 6 | 62.04 | 0.58 | 3.41 | 20.23 | 0.00 | 0.00 | 1.23 | 5.98 | 6.23 | 0.31 | 100 |
| Spectrum 143 | 6 | 58.83 | 0.98 | 3.52 | 21.19 | 0.07 | 0.61 | 0.71 | 7.51 | 5.97 | 0.61 | 100 |
| Spectrum 144 | 6 | 58.90 | 1.29 | 5.21 | 18.03 | 0.84 | 0.00 | 1.48 | 5.85 | 8.30 | 0.11 | 100 |
| Spectrum 145 | 6 | 56.63 | 0.66 | 4.56 | 21.41 | 0.15 | 0.21 | 1.44 | 8.00 | 6.74 | 0.21 | 100 |
| Spectrum 146 | 6 | 57.08 | 0.67 | 4.87 | 21.03 | 0.15 | 0.21 | 1.97 | 7.18 | 6.63 | 0.21 | 100 |
| Spectrum 147 | 6 | 56.25 | 0.59 | 4.95 | 21.59 | 0.22 | 0.00 | 1.65 | 7.32 | 7.01 | 0.42 | 100 |
| Spectrum 148 | 6 | 30.76 | 0.95 | 24.87 | 13.32 | 3.05 | 4.04 | 5.40 | 9.75 | 7.15 | 0.71 | 100 |
| Spectrum 149 | 6 | 60.16 | 0.41 | 2.49 | 20.72 | 0.00 | 0.82 | 1.55 | 7.52 | 6.03 | 0.31 | 100 |
| Spectrum 150 | 6 | 58.15 | 0.66 | 3.77 | 19.95 | 0.22 | 0.41 | 1.95 | 8.10 | 6.26 | 0.51 | 100 |
| Spectrum 151 | 6 | 59.45 | 0.90 | 3.52 | 20.57 | 0.29 | 0.81 | 1.29 | 7.51 | 5.26 | 0.41 | 100 |
| Spectrum 152 | 6 | 59.54 | 0.74 | 3.54 | 20.71 | 0.00 | 0.41 | 1.62 | 7.07 | 5.96 | 0.41 | 100 |
| Spectrum 153 | 6 | 59.47 | 0.49 | 3.40 | 20.08 | 0.29 | 1.03 | 1.75 | 7.32 | 5.86 | 0.31 | 100 |
| Spectrum 154 | 6 | 60.48 | 1.16 | 3.57 | 19.61 | 0.15 | 0.21 | 1.04 | 6.89 | 6.48 | 0.41 | 100 |
| Spectrum 155 | 6 | 61.52 | 0.99 | 3.47 | 19.69 | 0.00 | 0.31 | 0.97 | 6.83 | 6.01 | 0.20 | 100 |
| Spectrum 156 | 6 | 62.31 | 0.00 | 3.73 | 20.46 | 0.07 | 0.00 | 1.16 | 6.30 | 5.47 | 0.51 | 100 |
| Spectrum 157 | 6 | 60.89 | 1.23 | 2.99 | 20.58 | 0.22 | 0.10 | 0.78 | 6.58 | 6.22 | 0.41 | 100 |
| Spectrum 158 | 6 | 60.50 | 1.08 | 3.77 | 19.80 | 0.07 | 0.21 | 0.98 | 6.99 | 6.41 | 0.21 | 100 |
| Spectrum 159 | 6 | 60.76 | 0.83 | 3.79 | 19.50 | 0.37 | 0.00 | 1.31 | 6.65 | 6.59 | 0.21 | 100 |
| Spectrum 160 | 3 | 64.74 | 0.49 | 3.05 | 18.52 | 0.00 | 0.10 | 0.65 | 6.09 | 6.05 | 0.31 | 100 |
| Spectrum 161 | 3 | 64.88 | 0.41 | 2.76 | 18.60 | 0.15 | 0.00 | 0.45 | 6.31 | 6.04 | 0.41 | 100 |
| Spectrum 162 | 3 | 63.47 | 0.33 | 2.69 | 18.93 | 0.29 | 0.51 | 0.64 | 7.02 | 5.82 | 0.30 | 100 |
| Spectrum 163 | 3 | 62.82 | 0.73 | 2.48 | 19.26 | 1.01 | 0.30 | 0.45 | 6.89 | 5.65 | 0.41 | 100 |
| Spectrum 164 | 3 | 64.37 | 0.73 | 2.54 | 19.11 | 0.00 | 0.71 | 0.13 | 6.51 | 5.79 | 0.10 | 100 |
| Spectrum 165 | 3 | 64.45 | 0.65 | 2.61 | 18.97 | 0.00 | 0.91 | 0.45 | 6.27 | 5.59 | 0.10 | 100 |
| Spectrum 166 | 3 | 63.54 | 0.73 | 2.33 | 19.70 | 0.22 | 0.20 | 0.64 | 6.86 | 5.48 | 0.30 | 100 |
| Spectrum 167 | 3 | 64.96 | 0.65 | 2.41 | 19.13 | 0.00 | 0.00 | 0.45 | 6.40 | 5.80 | 0.20 | 100 |
| Spectrum 168 | 3 | 63.97 | 0.49 | 3.19 | 18.38 | 0.00 | 0.51 | 0.39 | 6.57 | 6.10 | 0.41 | 100 |
| Spectrum 169 | 3 | 64.30 | 0.73 | 2.88 | 18.93 | 0.00 | 0.00 | 0.77 | 6.98 | 5.02 | 0.40 | 100 |
| Spectrum 170 | 3 | 60.50 | 0.65 | 4.07 | 20.19 | 0.65 | 0.41 | 0.58 | 7.03 | 5.31 | 0.61 | 100 |
| Spectrum 171 | 3 | 61.38 | 0.90 | 2.69 | 20.17 | 0.00 | 0.10 | 0.90 | 7.38 | 5.87 | 0.61 | 100 |
| Spectrum 172 | 3 | 61.26 | 1.30 | 2.73 | 20.25 | 0.14 | 0.50 | 0.76 | 7.80 | 4.96 | 0.30 | 100 |
| Spectrum 173 | 3 | 61.28 | 1.05 | 3.08 | 20.01 | 0.14 | 0.30 | 0.45 | 7.92 | 5.16 | 0.60 | 100 |
| Spectrum 174 | 3 | 60.31 | 1.13 | 3.00 | 20.24 | 0.00 | 0.60 | 0.83 | 8.28 | 4.80 | 0.80 | 100 |
| Spectrum 175 | 3 | 63.22 | 0.40 | 2.66 | 19.38 | 0.00 | 0.10 | 0.19 | 7.92 | 5.61 | 0.50 | 100 |
| Spectrum 176 | 3 | 60.59 | 0.90 | 2.90 | 20.44 | 0.29 | 0.92 | 0.77 | 7.02 | 5.87 | 0.30 | 100 |
| Spectrum 177 | 3 | 63.14 | 0.89 | 1.99 | 19.82 | 0.65 | 0.61 | 0.51 | 6.50 | 5.48 | 0.40 | 100 |
| Spectrum 178 | 3 | 62.04 | 0.82 | 3.32 | 20.35 | 0.07 | 1.12 | 0.58 | 5.58 | 5.73 | 0.41 | 100 |
| Spectrum 179 | 3 | 61.73 | 0.82 | 3.10 | 20.44 | 0.00 | 0.81 | 0.90 | 6.18 | 5.61 | 0.41 | 100 |
| Spectrum 180 | 3 | 60.91 | 0.49 | 3.62 | 19.99 | 0.00 | 0.00 | 0.71 | 7.33 | 6.54 | 0.41 | 100 |
| Spectrum 181 | 3 | 61.61 | 0.57 | 3.67 | 20.16 | 0.00 | 0.00 | 0.52 | 6.69 | 6.26 | 0.51 | 100 |
| Spectrum 182 | 3 | 61.55 | 0.99 | 3.41 | 18.98 | 0.00 | 0.31 | 0.84 | 7.33 | 6.18 | 0.41 | 100 |
| Spectrum 183 | 3 | 62.95 | 1.25 | 4.28 | 19.37 | 0.15 | 0.52 | 1.57 | 4.06 | 5.65 | 0.21 | 100 |
| Spectrum 184 | 3 | 63.02 | 0.90 | 3.61 | 19.67 | 0.00 | 1.33 | 0.97 | 4.75 | 5.44 | 0.31 | 100 |
| Spectrum 185 | 3 | 60.79 | 1.25 | 4.91 | 19.77 | 0.00 | 0.52 | 1.90 | 5.05 | 5.61 | 0.21 | 100 |
| Spectrum 186 | 3 | 61.27 | 0.49 | 1.73 | 21.66 | 0.95 | 0.00 | 3.49 | 4.99 | 4.92 | 0.51 | 100 |
| Spectrum 187 | 3 | 60.87 | 0.00 | 3.32 | 20.63 | 0.00 | 0.51 | 4.06 | 5.95 | 4.25 | 0.41 | 100 |
| Spectrum 188 | 3 | 59.43 | 1.58 | 3.80 | 20.19 | 0.67 | 0.00 | 0.85 | 6.05 | 6.81 | 0.62 | 100 |
| Spectrum 189 | 3 | 59.32 | 0.81 | 3.43 | 20.11 | 0.36 | 0.71 | 0.90 | 8.56 | 5.29 | 0.51 | 100 |
| Spectrum 190 | 5 | 60.73 | 0.74 | 4.15 | 19.89 | 0.36 | 0.10 | 1.16 | 7.17 | 5.18 | 0.51 | 100 |
| Spectrum 191 | 5 | 61.91 | 0.65 | 3.03 | 19.90 | 0.22 | 0.00 | 1.35 | 6.89 | 5.14 | 0.91 | 100 |
| Spectrum 192 | 5 | 60.59 | 0.57 | 2.89 | 20.41 | 0.43 | 0.30 | 0.83 | 7.50 | 5.55 | 0.91 | 100 |
| Spectrum 193 | 5 | 60.76 | 0.66 | 3.68 | 19.56 | 0.44 | 0.41 | 1.10 | 7.19 | 5.50 | 0.72 | 100 |
| Spectrum 194 | 5 | 60.89 | 0.90 | 2.96 | 20.40 | 0.29 | 0.20 | 1.03 | 7.37 | 5.35 | 0.61 | 100 |
| Spectrum 195 | 5 | 62.01 | 0.33 | 3.37 | 19.77 | 0.36 | 0.30 | 1.09 | 7.01 | 5.35 | 0.41 | 100 |
| Spectrum 196 | 5 | 59.45 | 1.52 | 4.56 | 19.29 | 0.52 | 0.63 | 1.19 | 5.25 | 7.27 | 0.31 | 100 |
| Spectrum 197 | 5 | 61.12 | 1.34 | 4.79 | 19.46 | 0.00 | 0.21 | 1.31 | 4.70 | 6.25 | 0.83 | 100 |
| Spectrum 198 | 5 | 62.15 | 0.66 | 3.42 | 19.67 | 0.29 | 0.72 | 1.24 | 5.39 | 6.15 | 0.31 | 100 |
| Spectrum 199 | 5 | 62.04 | 1.08 | 3.72 | 19.51 | 0.59 | 0.21 | 1.24 | 5.17 | 6.23 | 0.21 | 100 |
| Spectrum 200 | 5 | 62.00 | 0.90 | 2.98 | 20.06 | 0.07 | 2.15 | 1.03 | 4.63 | 5.85 | 0.31 | 100 |
| Spectrum 201 | 5 | 62.75 | 0.82 | 2.56 | 19.34 | 0.07 | 2.85 | 1.16 | 4.73 | 5.21 | 0.51 | 100 |
| Spectrum 202 | 5 | 62.63 | 1.22 | 2.61 | 20.20 | 0.00 | 0.10 | 0.32 | 6.99 | 5.84 | 0.10 | 100 |
| Spectrum 203 | 5 | 62.09 | 0.57 | 2.33 | 19.79 | 0.29 | 0.40 | 0.64 | 8.17 | 5.52 | 0.20 | 100 |
| Spectrum 204 | 5 | 61.19 | 0.73 | 2.33 | 20.04 | 0.07 | 0.40 | 0.89 | 8.06 | 5.57 | 0.71 | 100 |
| Spectrum 205 | 5 | 62.27 | 0.65 | 2.19 | 19.56 | 0.14 | 0.10 | 0.83 | 8.42 | 5.53 | 0.30 | 100 |
| Spectrum 206 | 5 | 61.62 | 0.49 | 3.02 | 20.33 | 0.14 | 0.00 | 0.58 | 7.83 | 5.59 | 0.40 | 100 |
| Spectrum 207 | 5 | 58.62 | 0.66 | 3.46 | 21.27 | 0.51 | 0.51 | 0.65 | 7.78 | 6.15 | 0.41 | 100 |
| Spectrum 208 | 5 | 60.80 | 0.49 | 3.01 | 20.64 | 0.22 | 0.00 | 0.45 | 8.29 | 5.42 | 0.71 | 100 |
| Spectrum 209 | 5 | 60.32 | 0.49 | 3.36 | 20.47 | 0.07 | 0.20 | 0.51 | 8.32 | 5.44 | 0.81 | 100 |
| Spectrum 210 | 5 | 59.29 | 0.57 | 3.43 | 21.35 | 0.22 | 0.30 | 0.83 | 7.96 | 5.44 | 0.61 | 100 |
| Spectrum 211 | 5 | 60.79 | 0.49 | 3.02 | 19.83 | 0.29 | 0.10 | 0.70 | 8.79 | 5.48 | 0.51 | 100 |
| Spectrum 212 | 5 | 60.85 | 0.89 | 3.22 | 19.91 | 0.22 | 0.20 | 0.45 | 8.65 | 5.22 | 0.40 | 100 |
| Spectrum 213 | 5 | 60.07 | 0.57 | 3.00 | 20.60 | 0.36 | 0.00 | 0.89 | 9.11 | 5.00 | 0.40 | 100 |
| Spectrum 214 | 5 | 60.33 | 0.81 | 3.14 | 20.37 | 0.00 | 0.10 | 0.70 | 8.88 | 5.26 | 0.40 | 100 |
| Spectrum 215 | 5 | 60.88 | 1.45 | 4.44 | 18.92 | 0.00 | 0.00 | 0.87 | 4.79 | 8.50 | 0.16 | 100 |
| Spectrum 216 | 5 | 57.83 | 0.76 | 5.72 | 18.84 | 0.83 | 0.32 | 1.40 | 6.65 | 7.24 | 0.42 | 100 |
| Spectrum 217 | 5 | 58.85 | 0.25 | 5.32 | 19.14 | 0.00 | 0.73 | 0.66 | 6.99 | 7.74 | 0.31 | 100 |
| Spectrum 218 | 5 | 59.09 | 1.53 | 4.88 | 18.63 | 0.00 | 0.00 | 1.39 | 6.20 | 7.38 | 0.90 | 100 |
| Spectrum 219 | 5 | 60.43 | 1.34 | 4.02 | 19.25 | 0.00 | 0.00 | 0.46 | 5.70 | 7.37 | 1.43 | 100 |
| Spectrum 220 | 5 | 59.90 | 0.83 | 4.18 | 19.74 | 0.29 | 0.00 | 0.71 | 7.70 | 6.13 | 0.51 | 100 |
| Spectrum 221 | 5 | 61.92 | 0.49 | 2.64 | 19.47 | 0.00 | 0.00 | 1.17 | 7.20 | 6.48 | 0.61 | 100 |
| Spectrum 222 | 7 | 59.67 | 1.17 | 4.79 | 19.34 | 0.15 | 0.62 | 1.38 | 5.94 | 6.52 | 0.42 | 100 |
| Spectrum 223 | 7 | 60.33 | 0.92 | 4.57 | 18.93 | 0.22 | 0.83 | 1.44 | 5.93 | 6.30 | 0.52 | 100 |
| Spectrum 224 | 7 | 61.87 | 1.07 | 3.74 | 19.17 | 0.29 | 0.41 | 1.16 | 6.70 | 5.49 | 0.10 | 100 |
| Spectrum 225 | 7 | 60.72 | 0.91 | 3.71 | 19.71 | 0.22 | 0.31 | 1.37 | 6.27 | 6.37 | 0.41 | 100 |
| Spectrum 226 | 7 | 59.23 | 0.58 | 4.00 | 18.89 | 0.00 | 0.62 | 3.66 | 7.15 | 5.25 | 0.62 | 100 |
| Spectrum 227 | 7 | 58.89 | 1.50 | 5.40 | 19.64 | 0.52 | 0.10 | 1.50 | 6.53 | 5.40 | 0.52 | 100 |
| Spectrum 228 | 7 | 57.03 | 1.00 | 5.49 | 20.46 | 0.00 | 0.73 | 0.85 | 7.16 | 6.66 | 0.62 | 100 |
| Spectrum 229 | 7 | 59.92 | 1.53 | 5.46 | 18.30 | 0.15 | 0.42 | 1.27 | 4.80 | 7.61 | 0.53 | 100 |
| Spectrum 230 | 7 | 55.98 | 2.59 | 8.52 | 18.54 | 0.00 | 0.64 | 2.04 | 3.84 | 6.90 | 0.97 | 100 |
| Spectrum 231 | 7 | 59.29 | 1.48 | 5.07 | 20.22 | 0.37 | 0.00 | 1.36 | 6.35 | 4.84 | 1.02 | 100 |
| Spectrum 232 | 7 | 58.82 | 0.91 | 5.96 | 19.87 | 0.15 | 0.10 | 1.37 | 6.64 | 5.45 | 0.72 | 100 |
| Spectrum 233 | 7 | 58.07 | 0.59 | 7.24 | 19.48 | 0.00 | 1.05 | 2.84 | 4.86 | 4.94 | 0.94 | 100 |
| Spectrum 234 | 7 | 55.79 | 1.25 | 5.43 | 18.46 | 0.00 | 0.31 | 4.27 | 7.55 | 4.12 | 2.80 | 100 |
| Spectrum 235 | 7 | 56.99 | 1.18 | 5.32 | 18.06 | 0.37 | 0.00 | 4.36 | 6.85 | 4.67 | 2.19 | 100 |
| Spectrum 236 | 7 | 62.41 | 0.33 | 2.65 | 20.41 | 0.00 | 0.00 | 1.62 | 5.51 | 6.66 | 0.41 | 100 |
| Spectrum 237 | 7 | 61.46 | 0.50 | 2.51 | 20.41 | 0.15 | 0.00 | 2.14 | 5.88 | 6.35 | 0.62 | 100 |
| Spectrum 238 | 7 | 62.34 | 0.50 | 2.23 | 20.26 | 0.00 | 0.21 | 2.34 | 5.38 | 6.13 | 0.62 | 100 |
| Spectrum 239 | 7 | 60.44 | 0.25 | 3.61 | 21.13 | 0.15 | 0.00 | 3.76 | 3.73 | 6.29 | 0.63 | 100 |
| Spectrum 240 | 7 | 60.80 | 0.58 | 3.17 | 20.87 | 0.00 | 0.10 | 3.41 | 4.08 | 6.05 | 0.93 | 100 |
| Spectrum 241 | 7 | 60.35 | 0.42 | 3.32 | 21.06 | 0.00 | 0.31 | 3.36 | 3.97 | 6.38 | 0.83 | 100 |
| Spectrum 242 | 7 | 65.08 | 0.08 | 1.17 | 20.32 | 0.14 | 0.00 | 0.83 | 6.14 | 6.04 | 0.20 | 100 |
| Spectrum 243 | 7 | 63.55 | 0.41 | 1.24 | 20.05 | 0.22 | 0.61 | 0.84 | 6.41 | 6.38 | 0.30 | 100 |
| Spectrum 244 | 7 | 65.09 | 0.57 | 1.21 | 20.01 | 0.00 | 0.00 | 0.64 | 6.24 | 5.92 | 0.32 | 100 |
| Spectrum 245 | 7 | 61.58 | 0.73 | 2.67 | 21.99 | 0.22 | 0.00 | 2.29 | 6.12 | 4.20 | 0.20 | 100 |
| Spectrum 246 | 7 | 61.97 | 0.80 | 1.56 | 21.93 | 0.36 | 0.30 | 2.27 | 6.54 | 4.06 | 0.20 | 100 |
| Spectrum 247 | 7 | 60.97 | 0.82 | 3.13 | 19.21 | 0.44 | 0.21 | 1.30 | 7.45 | 5.97 | 0.51 | 100 |
| Spectrum 248 | 7 | 59.94 | 0.66 | 3.75 | 20.23 | 0.00 | 0.00 | 1.17 | 7.57 | 6.18 | 0.51 | 100 |
| Spectrum 249 | 7 | 61.05 | 0.58 | 3.68 | 19.83 | 0.15 | 0.10 | 1.04 | 7.20 | 6.07 | 0.31 | 100 |
| Spectrum 250 | 7 | 59.86 | 1.42 | 4.03 | 19.40 | 0.89 | 0.00 | 1.32 | 5.96 | 6.59 | 0.52 | 100 |
| Spectrum 251 | 7 | 55.10 | 0.42 | 5.83 | 19.16 | 0.00 | 5.70 | 0.95 | 8.56 | 3.38 | 0.90 | 100 |
| Spectrum 252 | 7 | 55.87 | 0.92 | 4.31 | 18.68 | 0.59 | 6.46 | 0.76 | 8.73 | 3.24 | 0.45 | 100 |
| Spectrum 253 | 7 | 55.20 | 0.84 | 4.85 | 18.70 | 0.15 | 6.22 | 0.89 | 9.05 | 3.33 | 0.79 | 100 |
| Spectrum 254 | 7 | 55.57 | 0.98 | 4.07 | 16.83 | 0.66 | 7.04 | 2.40 | 7.22 | 4.57 | 0.67 | 100 |
| Spectrum 255 | 7 | 56.40 | 1.19 | 3.87 | 15.88 | 0.00 | 7.27 | 2.60 | 7.37 | 4.74 | 0.67 | 100 |
| Spectrum 256 | 7 | 54.31 | 0.78 | 6.99 | 18.06 | 0.59 | 5.48 | 1.22 | 8.57 | 3.64 | 0.34 | 100 |
| Spectrum 257 | 7 | 53.51 | 0.99 | 6.80 | 18.16 | 0.00 | 6.17 | 1.15 | 8.63 | 3.57 | 1.02 | 100 |
| Spectrum 258 | 7 | 53.74 | 0.93 | 7.43 | 17.62 | 0.52 | 5.38 | 1.16 | 9.07 | 3.60 | 0.57 | 100 |
| Spectrum 259 | 7 | 57.65 | 0.63 | 4.06 | 17.73 | 0.22 | 6.20 | 1.20 | 8.06 | 3.58 | 0.67 | 100 |
| Spectrum 260 | 7 | 58.53 | 0.28 | 3.35 | 18.68 | 0.36 | 5.87 | 0.75 | 8.74 | 3.20 | 0.22 | 100 |
| Spectrum 261 | 7 | 51.81 | 0.86 | 8.95 | 16.85 | 0.00 | 7.82 | 0.97 | 8.85 | 3.45 | 0.46 | 100 |
| Spectrum 262 | 7 | 56.67 | 0.70 | 4.00 | 18.57 | 0.51 | 5.70 | 0.70 | 9.64 | 3.17 | 0.34 | 100 |
| Spectrum 263 | 7 | 59.72 | 0.84 | 4.50 | 20.25 | 0.15 | 0.00 | 1.58 | 6.07 | 6.57 | 0.33 | 100 |
| Spectrum 264 | 7 | 60.35 | 1.00 | 4.70 | 19.38 | 0.37 | 0.00 | 0.78 | 6.77 | 6.33 | 0.31 | 100 |
| Spectrum 265 | 7 | 61.20 | 0.07 | 5.12 | 19.24 | 0.08 | 0.11 | 0.79 | 6.66 | 6.61 | 0.12 | 100 |
| Spectrum 266 | 7 | 60.95 | 0.73 | 4.91 | 17.84 | 0.99 | 0.11 | 1.78 | 6.54 | 5.32 | 0.82 | 100 |
| Spectrum 267 | 7 | 63.66 | 0.36 | 4.68 | 17.63 | 0.07 | 0.32 | 0.32 | 6.91 | 4.90 | 1.15 | 100 |
| Spectrum 268 | 7 | 56.63 | 0.38 | 7.01 | 16.40 | 1.81 | 0.22 | 5.23 | 6.86 | 4.97 | 0.48 | 100 |
| Spectrum 269 | 7 | 58.72 | 0.29 | 3.74 | 19.16 | 0.38 | 0.64 | 4.93 | 9.14 | 2.66 | 0.34 | 100 |
| Spectrum 270 | 7 | 58.23 | 0.65 | 3.17 | 19.98 | 0.30 | 0.21 | 5.19 | 9.15 | 2.77 | 0.34 | 100 |
| Spectrum 271 | 7 | 56.64 | 1.32 | 6.16 | 17.25 | 0.46 | 0.65 | 5.09 | 8.68 | 2.93 | 0.82 | 100 |
| Spectrum 272 | 7 | 57.04 | 1.17 | 4.76 | 19.03 | 0.69 | 0.11 | 5.48 | 7.93 | 3.09 | 0.70 | 100 |
| Spectrum 273 | 7 | 57.89 | 0.64 | 3.94 | 19.32 | 0.37 | 0.42 | 4.85 | 10.09 | 2.12 | 0.34 | 100 |
| Spectrum 274 | 7 | 58.41 | 0.43 | 3.20 | 19.27 | 0.22 | 0.21 | 4.55 | 10.98 | 2.16 | 0.57 | 100 |
| Spectrum 275 | 7 | 59.33 | 0.71 | 3.79 | 19.14 | 0.52 | 0.11 | 4.70 | 9.45 | 1.80 | 0.46 | 100 |
| Spectrum 276 | 7 | 51.81 | 0.86 | 8.95 | 16.85 | 0.00 | 7.82 | 0.97 | 8.85 | 3.45 | 0.46 | 100 |
| Spectrum 277 | 7 | 56.67 | 0.70 | 4.00 | 18.57 | 0.51 | 5.70 | 0.70 | 9.64 | 3.17 | 0.34 | 100 |
| Spectrum 278 | 7 | 60.23 | 0.74 | 4.75 | 19.03 | 0.15 | 0.33 | 1.61 | 6.24 | 6.92 | 0.00 | 100 |
| Spectrum 279 | 7 | 60.82 | 0.88 | 4.94 | 18.19 | 0.38 | 0.00 | 0.80 | 6.96 | 6.66 | 0.35 | 100 |
| Spectrum 280 | 7 | 54.99 | 0.73 | 4.37 | 18.31 | 1.06 | 0.32 | 6.69 | 10.37 | 2.10 | 1.05 | 100 |
| Spectrum 281 | 7 | 57.25 | 1.85 | 6.23 | 18.76 | 0.08 | 0.11 | 5.08 | 8.78 | 1.76 | 0.12 | 100 |
| Spectrum 282 | 7 | 56.01 | 0.67 | 5.77 | 18.83 | 1.01 | 0.11 | 5.67 | 8.87 | 2.83 | 0.24 | 100 |
| Spectrum 283 | 7 | 63.25 | 0.14 | 2.38 | 18.47 | 0.08 | 0.11 | 0.78 | 7.79 | 6.08 | 0.92 | 100 |

**2**.2 Orotava volcanic glass compositions in wt%

| Name | Sub-unit | SiO_2_ | TiO_2_ | Fe_2_O_3_T | Al_2_O_3_ | MnO | MgO | CaO | Na_2_O | K_2_O | P_2_O_5_ | Total |
| --- | --- | --- | --- | --- | --- | --- | --- | --- | --- | --- | --- | --- |
| Spectrum01 | 1 | 58.78 | 1.20 | 6.86 | 17.39 | 0.00 | 1.28 | 2.23 | 5.12 | 6.49 | 0.65 | 100 |
| Spectrum02 | 1 | 57.80 | 0.67 | 6.87 | 17.51 | 1.27 | 1.05 | 1.71 | 6.52 | 6.61 | 0.00 | 100 |
| Spectrum03 | 1 | 58.89 | 1.41 | 6.40 | 17.88 | 0.15 | 1.27 | 1.18 | 6.13 | 6.69 | 0.00 | 100 |
| Spectrum04 | 1 | 51.21 | 1.49 | 13.37 | 17.67 | 0.08 | 0.61 | 2.68 | 5.11 | 7.46 | 0.34 | 100 |
| Spectrum05 | 1 | 53.56 | 1.62 | 10.59 | 17.51 | 0.00 | 1.79 | 2.23 | 4.91 | 7.46 | 0.33 | 100 |
| Spectrum06 | 1 | 54.94 | 1.85 | 9.19 | 17.64 | 0.61 | 0.96 | 2.57 | 4.43 | 7.58 | 0.22 | 100 |
| Spectrum07 | 1 | 53.12 | 1.24 | 10.19 | 16.37 | 0.92 | 0.84 | 2.58 | 5.89 | 7.85 | 1.00 | 100 |
| Spectrum08 | 1 | 47.67 | 3.43 | 10.78 | 15.99 | 0.54 | 5.56 | 9.50 | 3.51 | 2.01 | 1.01 | 100 |
| Spectrum09 | 1 | 47.67 | 3.81 | 10.31 | 15.83 | 0.46 | 6.15 | 9.81 | 3.26 | 1.58 | 1.12 | 100 |
| Spectrum10 | 1 | 56.99 | 0.00 | 9.62 | 16.88 | 1.45 | 0.36 | 1.15 | 4.90 | 8.65 | 0.00 | 100 |
| Spectrum11 | 1 | 57.62 | 1.23 | 9.10 | 15.05 | 0.54 | 0.00 | 1.08 | 6.81 | 8.58 | 0.00 | 100 |
| Spectrum12 | 1 | 57.54 | 0.92 | 6.92 | 16.51 | 0.91 | 0.47 | 1.00 | 5.45 | 9.18 | 1.10 | 100 |
| Spectrum13 | 1 | 44.42 | 4.32 | 14.90 | 14.28 | 0.32 | 4.35 | 9.90 | 3.61 | 2.06 | 1.84 | 100 |
| Spectrum14 | 1 | 49.35 | 0.60 | 3.22 | 27.38 | 0.22 | 0.35 | 14.68 | 3.27 | 0.18 | 0.75 | 100 |
| Spectrum15 | 1 | 49.84 | 0.82 | 3.51 | 27.23 | 0.07 | 0.82 | 13.74 | 3.38 | 0.59 | 0.00 | 100 |
| Spectrum16 | 1 | 56.35 | 1.56 | 4.97 | 20.22 | 2.07 | 0.00 | 1.18 | 6.01 | 6.57 | 1.07 | 100 |
| Spectrum17 | 1 | 56.95 | 0.82 | 7.03 | 17.97 | 0.00 | 0.81 | 1.30 | 8.55 | 6.56 | 0.00 | 100 |
| Spectrum18 | 1 | 57.96 | 2.17 | 6.85 | 17.81 | 0.74 | 0.00 | 0.26 | 6.85 | 6.83 | 0.54 | 100 |
| Spectrum19 | 1 | 59.06 | 0.89 | 5.11 | 18.77 | 0.52 | 0.46 | 1.04 | 6.69 | 7.25 | 0.21 | 100 |
| Spectrum20 | 1 | 55.80 | 1.05 | 7.79 | 18.09 | 1.42 | 0.23 | 1.64 | 8.49 | 5.50 | 0.00 | 100 |
| Spectrum21 | 1 | 57.46 | 0.67 | 7.39 | 17.76 | 0.00 | 1.16 | 1.24 | 7.64 | 6.69 | 0.00 | 100 |
| Spectrum22 | 1 | 55.55 | 1.15 | 10.51 | 17.87 | 0.23 | 1.19 | 1.34 | 4.75 | 7.41 | 0.00 | 100 |
| Spectrum23 | 1 | 57.09 | 2.81 | 7.47 | 18.48 | 0.00 | 1.42 | 2.00 | 3.66 | 7.06 | 0.00 | 100 |
| Spectrum24 | 1 | 48.87 | 3.19 | 12.03 | 15.48 | 0.00 | 5.44 | 9.28 | 3.63 | 1.52 | 0.56 | 100 |
| Spectrum25 | 1 | 49.86 | 3.19 | 11.43 | 15.71 | 0.00 | 5.68 | 9.41 | 3.02 | 1.70 | 0.00 | 100 |
| Spectrum26 | 1 | 51.95 | 2.02 | 11.71 | 17.77 | 0.00 | 0.84 | 2.86 | 5.31 | 7.09 | 0.45 | 100 |
| Spectrum27 | 1 | 52.89 | 0.64 | 15.09 | 13.62 | 0.08 | 3.01 | 3.11 | 1.50 | 9.82 | 0.23 | 100 |
| Spectrum28 | 1 | 52.22 | 2.87 | 11.43 | 14.36 | 0.00 | 1.98 | 3.64 | 3.22 | 9.47 | 0.80 | 100 |
| Spectrum29 | 1 | 51.46 | 1.53 | 13.47 | 13.49 | 1.36 | 2.00 | 4.31 | 3.13 | 9.13 | 0.12 | 100 |
| Spectrum30 | 2 | 49.24 | 0.68 | 2.44 | 33.54 | 0.00 | 1.17 | 0.39 | 0.47 | 12.08 | 0.00 | 100 |
| Spectrum31 | 2 | 52.03 | 0.85 | 5.86 | 17.42 | 0.29 | 1.58 | 4.27 | 3.76 | 6.12 | 7.82 | 100 |
| Spectrum32 | 2 | 56.75 | 1.40 | 6.81 | 17.08 | 0.15 | 1.68 | 2.91 | 1.93 | 2.39 | 8.91 | 100 |
| Spectrum33 | 2 | 51.67 | 0.98 | 9.12 | 16.17 | 0.44 | 1.68 | 2.97 | 5.42 | 2.44 | 9.11 | 100 |
| Spectrum34 | 2 | 49.68 | 1.43 | 7.44 | 17.99 | 0.37 | 0.23 | 2.64 | 6.01 | 4.82 | 9.39 | 100 |
| Spectrum35 | 2 | 57.23 | 1.19 | 4.29 | 18.85 | 0.59 | 0.34 | 1.65 | 4.11 | 3.96 | 7.79 | 100 |
| Spectrum36 | 2 | 54.03 | 0.85 | 6.69 | 17.89 | 0.67 | 0.45 | 0.71 | 7.20 | 3.21 | 8.29 | 100 |
| Spectrum37 | 2 | 50.54 | 1.90 | 12.89 | 14.84 | 1.89 | 5.52 | 2.84 | 1.72 | 7.53 | 0.34 | 100 |
| Spectrum38 | 2 | 51.62 | 1.41 | 12.71 | 14.47 | 1.09 | 5.35 | 3.16 | 2.19 | 6.98 | 1.01 | 100 |
| Spectrum39 | 2 | 51.99 | 0.49 | 15.04 | 14.82 | 0.00 | 1.90 | 4.15 | 0.38 | 11.22 | 0.00 | 100 |
| Spectrum40 | 2 | 54.09 | 2.15 | 10.97 | 14.36 | 0.24 | 1.86 | 3.29 | 2.36 | 10.34 | 0.34 | 100 |
| Spectrum41 | 2 | 55.30 | 0.00 | 15.21 | 11.88 | 0.00 | 1.63 | 3.55 | 2.14 | 10.30 | 0.00 | 100 |
| Spectrum42 | 2 | 62.55 | 0.44 | 4.37 | 17.94 | 0.00 | 0.23 | 1.49 | 5.62 | 7.15 | 0.21 | 100 |
| Spectrum43 | 3 | 45.35 | 6.63 | 18.46 | 12.03 | 0.90 | 3.05 | 9.27 | 1.27 | 1.86 | 1.18 | 100 |
| Spectrum44 | 3 | 46.86 | 6.69 | 17.69 | 12.25 | 0.08 | 2.92 | 9.38 | 1.40 | 1.91 | 0.82 | 100 |
| Spectrum45 | 3 | 46.13 | 7.22 | 17.70 | 10.88 | 0.16 | 2.93 | 10.22 | 1.91 | 1.67 | 1.18 | 100 |
| Spectrum46 | 3 | 95.24 | 0.83 | 0.86 | 1.76 | 0.42 | 0.00 | 0.24 | 0.00 | 0.65 | 0.00 | 100 |
| Spectrum47 | 3 | 56.23 | 1.69 | 7.92 | 16.98 | 0.92 | 0.84 | 1.35 | 4.65 | 8.76 | 0.66 | 100 |
| Spectrum48 | 3 | 58.22 | 1.31 | 6.75 | 16.52 | 0.92 | 0.48 | 1.62 | 4.42 | 9.55 | 0.22 | 100 |
| Spectrum49 | 3 | 45.05 | 4.02 | 18.64 | 15.22 | 0.08 | 4.12 | 8.60 | 2.25 | 1.57 | 0.46 | 100 |
| Spectrum50 | 3 | 43.94 | 5.04 | 18.87 | 16.55 | 0.24 | 3.73 | 7.72 | 1.99 | 1.12 | 0.80 | 100 |
| Spectrum51 | 3 | 59.18 | 0.82 | 7.29 | 17.92 | 0.52 | 0.69 | 1.43 | 6.34 | 5.50 | 0.32 | 100 |
| Spectrum52 | 3 | 59.09 | 0.81 | 6.86 | 18.36 | 0.15 | 0.92 | 1.49 | 6.44 | 5.67 | 0.21 | 100 |
| Spectrum53 | 3 | 59.11 | 0.74 | 7.96 | 17.99 | 0.30 | 0.93 | 1.31 | 5.90 | 5.76 | 0.00 | 100 |
| Spectrum54 | 3 | 55.49 | 1.22 | 8.70 | 18.86 | 1.30 | 0.59 | 1.01 | 4.64 | 8.19 | 0.00 | 100 |
| Spectrum55 | 3 | 58.58 | 1.12 | 6.35 | 19.96 | 0.07 | 0.70 | 1.11 | 4.07 | 7.71 | 0.32 | 100 |
| Spectrum56 | 3 | 59.42 | 1.92 | 6.42 | 18.68 | 0.44 | 1.03 | 1.62 | 5.40 | 4.85 | 0.21 | 100 |
| Spectrum57 | 3 | 60.47 | 1.47 | 7.02 | 19.26 | 0.00 | 1.14 | 1.68 | 4.34 | 4.42 | 0.21 | 100 |
| Spectrum58 | 3 | 60.01 | 1.54 | 6.25 | 19.40 | 0.37 | 0.91 | 1.74 | 4.69 | 4.66 | 0.42 | 100 |
| Spectrum59 | 3 | 57.80 | 1.59 | 5.50 | 18.90 | 0.15 | 0.35 | 2.06 | 5.06 | 8.58 | 0.00 | 100 |
| Spectrum60 | 3 | 57.72 | 1.06 | 7.08 | 17.92 | 1.13 | 0.59 | 1.73 | 5.41 | 7.15 | 0.22 | 100 |
| Spectrum61 | 3 | 59.21 | 1.12 | 6.35 | 18.06 | 0.60 | 0.81 | 0.98 | 5.57 | 7.30 | 0.00 | 100 |
| Spectrum62 | 3 | 57.83 | 0.83 | 7.05 | 18.68 | 0.08 | 0.23 | 1.85 | 5.97 | 7.48 | 0.00 | 100 |
| Spectrum63 | 3 | 52.01 | 2.44 | 15.23 | 14.52 | 2.43 | 1.83 | 1.38 | 4.15 | 6.02 | 0.00 | 100 |
| Spectrum64 | 3 | 52.93 | 2.85 | 16.54 | 14.61 | 0.00 | 0.86 | 1.04 | 3.32 | 7.17 | 0.68 | 100 |
| Spectrum65 | 3 | 59.80 | 1.28 | 6.67 | 17.78 | 0.00 | 0.93 | 1.12 | 4.55 | 7.45 | 0.43 | 100 |
| Spectrum66 | 3 | 59.38 | 1.12 | 6.26 | 18.25 | 0.00 | 0.81 | 1.24 | 5.91 | 6.93 | 0.11 | 100 |
| Spectrum67 | 3 | 59.34 | 1.12 | 5.55 | 18.47 | 0.74 | 0.81 | 1.37 | 5.21 | 6.75 | 0.64 | 100 |
| Spectrum68 | 4 | 52.67 | 2.56 | 12.77 | 14.42 | 0.24 | 0.62 | 1.76 | 3.73 | 10.64 | 0.58 | 100 |
| Spectrum69 | 4 | 51.51 | 2.01 | 13.19 | 14.97 | 0.00 | 0.62 | 2.25 | 4.37 | 10.85 | 0.23 | 100 |
| Spectrum70 | 4 | 53.47 | 1.51 | 9.25 | 17.17 | 0.63 | 0.86 | 1.81 | 3.08 | 12.22 | 0.00 | 100 |
| Spectrum71 | 4 | 52.50 | 0.00 | 10.65 | 19.44 | 1.07 | 0.96 | 2.36 | 5.02 | 7.45 | 0.55 | 100 |
| Spectrum72 | 4 | 53.23 | 2.22 | 8.99 | 19.22 | 0.46 | 0.71 | 1.28 | 5.83 | 8.07 | 0.00 | 100 |
| Spectrum73 | 4 | 52.50 | 0.00 | 10.65 | 19.44 | 1.07 | 0.96 | 2.36 | 5.02 | 7.45 | 0.55 | 100 |
| Spectrum74 | 4 | 53.23 | 2.22 | 8.99 | 19.22 | 0.46 | 0.71 | 1.28 | 5.83 | 8.07 | 0.00 | 100 |
| Spectrum75 | 4 | 53.49 | 2.38 | 10.13 | 18.70 | 0.08 | 0.96 | 1.42 | 4.66 | 7.75 | 0.44 | 100 |
| Spectrum76 | 4 | 61.12 | 0.87 | 3.69 | 19.21 | 0.51 | 0.45 | 1.21 | 7.13 | 5.80 | 0.00 | 100 |
| Spectrum77 | 4 | 61.10 | 0.58 | 4.10 | 19.43 | 0.00 | 0.57 | 0.89 | 7.13 | 6.20 | 0.00 | 100 |
| Spectrum78 | 4 | 60.56 | 0.66 | 3.55 | 20.01 | 0.36 | 0.57 | 1.15 | 7.24 | 5.92 | 0.00 | 100 |
| Spectrum79 | 4 | 60.12 | 0.80 | 4.82 | 19.07 | 0.29 | 0.45 | 1.09 | 6.94 | 5.89 | 0.53 | 100 |
| Spectrum80 | 4 | 63.53 | 0.29 | 3.64 | 17.36 | 0.80 | 0.46 | 0.96 | 6.26 | 6.70 | 0.00 | 100 |
| Spectrum81 | 4 | 61.95 | 0.73 | 3.28 | 18.35 | 0.58 | 0.79 | 0.96 | 6.02 | 6.39 | 0.94 | 100 |
| Spectrum82 | 4 | 62.84 | 0.95 | 3.85 | 17.46 | 0.29 | 0.57 | 1.03 | 6.60 | 6.41 | 0.00 | 100 |
| Spectrum83 | 4 | 48.76 | 3.54 | 11.01 | 16.39 | 0.00 | 5.73 | 7.96 | 3.70 | 1.80 | 1.10 | 100 |
| Spectrum84 | 4 | 49.76 | 3.61 | 10.57 | 16.27 | 0.15 | 5.25 | 8.02 | 3.46 | 1.68 | 1.21 | 100 |
| Spectrum85 | 4 | 49.66 | 3.70 | 10.01 | 15.82 | 0.23 | 5.75 | 8.38 | 3.95 | 2.05 | 0.44 | 100 |
| Spectrum86 | 4 | 56.72 | 1.55 | 11.39 | 15.17 | 0.62 | 1.08 | 2.72 | 3.97 | 6.78 | 0.00 | 100 |
| Spectrum87 | 4 | 56.24 | 2.25 | 11.11 | 15.81 | 0.15 | 0.72 | 2.04 | 4.34 | 7.33 | 0.00 | 100 |
| Spectrum88 | 4 | 65.51 | 0.00 | 3.41 | 16.91 | 0.36 | 0.23 | 0.58 | 5.77 | 6.60 | 0.63 | 100 |
| Spectrum89 | 4 | 65.06 | 0.36 | 4.30 | 17.69 | 0.00 | 0.11 | 0.64 | 5.76 | 5.96 | 0.10 | 100 |
| Spectrum90 | 4 | 64.43 | 0.22 | 3.97 | 17.28 | 0.36 | 0.34 | 0.83 | 6.24 | 6.33 | 0.00 | 100 |
| Spectrum91 | 4 | 64.28 | 0.81 | 4.20 | 16.78 | 0.36 | 0.57 | 1.48 | 5.47 | 5.84 | 0.21 | 100 |
| Spectrum92 | 4 | 56.14 | 1.66 | 5.26 | 17.88 | 0.23 | 0.82 | 5.36 | 4.46 | 5.60 | 2.60 | 100 |
| Spectrum93 | 4 | 55.53 | 1.06 | 6.23 | 17.49 | 0.15 | 1.53 | 5.72 | 4.01 | 5.57 | 2.72 | 100 |
| Spectrum94 | 4 | 62.23 | 0.74 | 3.51 | 18.81 | 0.59 | 0.34 | 1.35 | 5.48 | 6.95 | 0.00 | 100 |
| Spectrum95 | 4 | 60.17 | 1.74 | 9.88 | 14.31 | 0.15 | 0.35 | 0.86 | 6.34 | 6.20 | 0.00 | 100 |
| Spectrum96 | 4 | 59.54 | 1.67 | 9.04 | 14.71 | 0.60 | 0.35 | 1.20 | 6.00 | 6.57 | 0.33 | 100 |
| Spectrum97 | 4 | 62.55 | 0.44 | 4.37 | 17.94 | 0.00 | 0.23 | 1.49 | 5.62 | 7.15 | 0.21 | 100 |
| Spectrum98 | 4 | 59.85 | 1.27 | 5.19 | 18.11 | 0.52 | 0.69 | 1.18 | 5.67 | 7.21 | 0.32 | 100 |
| Spectrum99 | 4 | 61.88 | 1.10 | 3.37 | 18.48 | 0.00 | 0.80 | 1.29 | 5.72 | 7.25 | 0.11 | 100 |
| Spectrum100 | 4 | 61.08 | 0.89 | 4.83 | 17.97 | 0.52 | 0.69 | 0.98 | 5.32 | 7.73 | 0.00 | 100 |
| Spectrum101 | 4 | 61.01 | 1.11 | 3.46 | 18.66 | 0.00 | 0.46 | 2.01 | 6.31 | 6.98 | 0.00 | 100 |
| Spectrum102 | 4 | 59.39 | 1.18 | 5.56 | 17.93 | 0.15 | 0.46 | 1.42 | 7.33 | 6.05 | 0.53 | 100 |
| Spectrum103 | 4 | 59.47 | 2.27 | 8.39 | 14.12 | 0.00 | 0.82 | 1.53 | 6.59 | 6.81 | 0.00 | 100 |
| Spectrum104 | 4 | 57.18 | 1.21 | 5.34 | 17.77 | 0.00 | 1.06 | 5.17 | 3.40 | 5.72 | 3.15 | 100 |
| Spectrum105 | 4 | 64.52 | 0.68 | 2.44 | 16.83 | 0.00 | 0.35 | 1.78 | 3.62 | 9.79 | 0.00 | 100 |
| Spectrum106 | 4 | 55.53 | 1.06 | 6.23 | 17.49 | 0.15 | 1.53 | 5.72 | 4.01 | 5.57 | 2.72 | 100 |
| Spectrum107 | 4 | 57.18 | 1.21 | 5.34 | 17.77 | 0.00 | 1.06 | 5.17 | 3.40 | 5.72 | 3.15 | 100 |
| Spectrum108 | 5 | 64.52 | 0.68 | 2.44 | 16.83 | 0.00 | 0.35 | 1.78 | 3.62 | 9.79 | 0.00 | 100 |
| Spectrum109 | 5 | 64.82 | 0.00 | 3.20 | 17.62 | 0.22 | 0.12 | 1.44 | 3.82 | 8.78 | 0.00 | 100 |
| Spectrum110 | 5 | 64.50 | 0.67 | 3.07 | 16.65 | 0.30 | 0.35 | 1.57 | 3.84 | 8.94 | 0.11 | 100 |
| Spectrum111 | 5 | 63.95 | 0.30 | 3.56 | 17.53 | 0.00 | 0.46 | 1.24 | 4.05 | 8.91 | 0.00 | 100 |
| Spectrum112 | 5 | 64.10 | 0.30 | 2.85 | 16.63 | 0.00 | 0.35 | 1.90 | 4.30 | 8.93 | 0.64 | 100 |
| Spectrum113 | 5 | 58.16 | 0.55 | 4.31 | 17.96 | 0.23 | 0.85 | 2.05 | 2.42 | 13.14 | 0.34 | 100 |
| Spectrum114 | 5 | 60.86 | 0.85 | 2.59 | 17.48 | 0.39 | 0.96 | 1.90 | 1.92 | 13.06 | 0.00 | 100 |
| Spectrum115 | 5 | 60.82 | 0.69 | 2.73 | 17.31 | 0.00 | 0.96 | 2.23 | 1.80 | 12.79 | 0.67 | 100 |
| Spectrum116 | 5 | 54.15 | 1.71 | 9.12 | 16.56 | 1.55 | 1.45 | 4.29 | 3.38 | 6.68 | 1.12 | 100 |
| Spectrum117 | 5 | 54.91 | 1.86 | 11.59 | 17.22 | 0.00 | 0.36 | 3.73 | 4.09 | 5.92 | 0.33 | 100 |
| Spectrum118 | 5 | 56.31 | 1.01 | 7.30 | 15.15 | 0.78 | 1.09 | 6.02 | 3.52 | 7.92 | 0.90 | 100 |
| Spectrum119 | 5 | 57.49 | 1.32 | 7.13 | 15.47 | 0.00 | 0.24 | 6.55 | 4.47 | 7.23 | 0.11 | 100 |
| Spectrum120 | 5 | 56.37 | 0.87 | 8.12 | 15.29 | 0.78 | 0.49 | 7.46 | 3.18 | 7.44 | 0.00 | 100 |
| Spectrum121 | 5 | 60.57 | 1.30 | 6.14 | 15.48 | 0.00 | 0.24 | 4.37 | 3.81 | 7.54 | 0.55 | 100 |
| Spectrum122 | 5 | 58.58 | 0.77 | 6.55 | 16.31 | 0.00 | 0.72 | 4.12 | 3.71 | 8.79 | 0.44 | 100 |
| Spectrum123 | 5 | 61.26 | 0.59 | 4.09 | 17.83 | 0.29 | 0.69 | 2.78 | 6.31 | 6.17 | 0.00 | 100 |
| Spectrum124 | 5 | 61.36 | 0.59 | 3.87 | 18.17 | 0.51 | 0.34 | 2.52 | 6.08 | 6.22 | 0.32 | 100 |
| Spectrum125 | 5 | 62.36 | 0.96 | 4.38 | 17.31 | 0.22 | 0.69 | 2.99 | 5.18 | 5.90 | 0.00 | 100 |
| Spectrum126 | 5 | 61.25 | 0.74 | 4.60 | 17.22 | 0.30 | 0.81 | 2.93 | 5.88 | 5.85 | 0.43 | 100 |
| Spectrum127 | 5 | 52.28 | 1.82 | 9.75 | 16.25 | 1.65 | 0.49 | 3.40 | 3.81 | 9.40 | 1.14 | 100 |
| Spectrum128 | 5 | 52.85 | 1.34 | 12.37 | 15.59 | 0.00 | 0.98 | 3.05 | 4.42 | 9.07 | 0.34 | 100 |
| Spectrum129 | 5 | 51.26 | 2.13 | 10.94 | 16.10 | 0.87 | 0.37 | 3.60 | 5.40 | 8.76 | 0.57 | 100 |
| Spectrum130 | 5 | 61.76 | 0.38 | 3.55 | 17.01 | 0.60 | 0.59 | 4.39 | 3.42 | 8.30 | 0.00 | 100 |
| Spectrum131 | 5 | 61.43 | 0.53 | 3.89 | 16.32 | 0.00 | 0.94 | 4.37 | 4.10 | 7.66 | 0.76 | 100 |
| Spectrum132 | 5 | 62.60 | 0.61 | 4.05 | 16.01 | 0.15 | 0.59 | 4.31 | 4.00 | 7.68 | 0.00 | 100 |
| Spectrum133 | 5 | 51.32 | 3.35 | 8.23 | 15.63 | 0.95 | 1.74 | 15.20 | 1.24 | 1.87 | 0.46 | 100 |
| Spectrum134 | 5 | 49.89 | 3.62 | 9.23 | 15.14 | 1.44 | 1.50 | 14.98 | 1.13 | 2.14 | 0.93 | 100 |
| Spectrum135 | 5 | 52.32 | 3.57 | 19.13 | 11.78 | 0.31 | 7.24 | 2.66 | 0.36 | 1.52 | 1.12 | 100 |
| Spectrum136 | 5 | 46.94 | 2.95 | 12.30 | 18.02 | 0.00 | 5.91 | 8.03 | 3.26 | 2.49 | 0.11 | 100 |
| Spectrum137 | 5 | 46.71 | 3.39 | 12.78 | 17.16 | 0.39 | 4.04 | 10.02 | 3.43 | 1.97 | 0.11 | 100 |
| Spectrum138 | 5 | 60.01 | 0.87 | 3.85 | 19.69 | 0.53 | 0.46 | 1.24 | 7.86 | 5.49 | 0.00 | 100 |
| Spectrum139 | 5 | 60.00 | 0.58 | 4.29 | 19.92 | 0.00 | 0.58 | 0.92 | 7.86 | 5.86 | 0.00 | 100 |
| Spectrum140 | 5 | 61.75 | 0.79 | 3.42 | 18.69 | 0.07 | 0.45 | 1.07 | 7.58 | 5.88 | 0.31 | 100 |
| Spectrum141 | 5 | 61.65 | 0.36 | 3.18 | 19.42 | 0.36 | 0.56 | 0.95 | 7.64 | 5.88 | 0.00 | 100 |
| Spectrum142 | 5 | 60.67 | 1.90 | 7.62 | 13.69 | 0.45 | 0.59 | 1.40 | 6.26 | 7.42 | 0.00 | 100 |
| Spectrum143 | 5 | 61.58 | 0.58 | 2.91 | 18.92 | 0.14 | 0.68 | 1.34 | 7.67 | 6.07 | 0.10 | 100 |
| Spectrum144 | 5 | 61.41 | 0.58 | 2.84 | 19.36 | 0.22 | 0.79 | 0.95 | 7.44 | 6.40 | 0.00 | 100 |
| Spectrum145 | 5 | 61.93 | 0.65 | 2.07 | 19.06 | 0.65 | 0.45 | 0.82 | 7.86 | 5.99 | 0.52 | 100 |
| Spectrum146 | 5 | 61.53 | 0.51 | 2.84 | 19.25 | 0.36 | 0.34 | 1.15 | 7.67 | 6.35 | 0.00 | 100 |
| Spectrum147 | 5 | 60.71 | 0.80 | 3.60 | 18.69 | 0.07 | 0.68 | 0.89 | 7.90 | 6.24 | 0.42 | 100 |
| Spectrum148 | 5 | 58.57 | 0.95 | 5.03 | 18.47 | 0.29 | 0.68 | 1.35 | 8.41 | 5.94 | 0.32 | 100 |
| Spectrum149 | 5 | 59.98 | 0.81 | 3.64 | 18.31 | 0.66 | 0.80 | 1.22 | 7.87 | 6.71 | 0.00 | 100 |
| Spectrum150 | 5 | 59.44 | 0.65 | 3.70 | 20.50 | 0.38 | 0.58 | 1.18 | 7.98 | 5.59 | 0.00 | 100 |
| Spectrum151 | 5 | 47.44 | 2.63 | 11.62 | 15.91 | 0.23 | 5.18 | 8.84 | 5.42 | 2.06 | 0.67 | 100 |

**2**.3 Rogues de Garcia volcanic glass compositions in wt%

| Name | Sub-unit | SiO_2_ | TiO_2_ | Fe_2_O_3_T | Al_2_O_3_ | MnO | MgO | CaO | Na_2_O | K_2_O | P_2_O_5_ | Total |
| --- | --- | --- | --- | --- | --- | --- | --- | --- | --- | --- | --- | --- |
| Spectrum01 | 1 | 47.43 | 1.76 | 3.72 | 6.45 | 0.12 | 13.98 | 20.15 | 2.31 | 4.01 | 0.07 | 100 |
| Spectrum02 | 1 | 47.34 | 1.42 | 3.78 | 6.65 | 0.03 | 13.76 | 20.31 | 2.41 | 4.09 | 0.21 | 100 |
| Spectrum03 | 1 | 48.41 | 1.64 | 3.22 | 6.49 | 0.08 | 13.78 | 19.66 | 2.42 | 4.06 | 0.23 | 100 |
| Spectrum04 | 1 | 47.89 | 1.98 | 3.77 | 5.66 | 0.11 | 13.42 | 20.37 | 2.45 | 4.12 | 0.23 | 100 |
| Spectrum05 | 1 | 47.55 | 1.57 | 4.37 | 6.02 | 0.11 | 13.98 | 19.49 | 2.85 | 4.06 | 0.00 | 100 |
| Spectrum06 | 1 | 45.34 | 1.54 | 3.98 | 6.01 | 1.20 | 14.01 | 19.53 | 3.01 | 5.32 | 0.06 | 100 |
| Spectrum07 | 1 | 45.41 | 1.45 | 4.12 | 6.43 | 0.04 | 14.23 | 19.54 | 3.12 | 5.62 | 0.04 | 100 |
| Spectrum08 | 1 | 45.87 | 1.66 | 4.32 | 6.12 | 0.00 | 13.97 | 19.50 | 3.24 | 5.32 | 0.00 | 100 |
| Spectrum09 | 1 | 45.51 | 1.87 | 3.96 | 6.14 | 1.10 | 13.78 | 18.98 | 3.01 | 5.24 | 0.41 | 100 |
| Spectrum10 | 1 | 45.61 | 1.78 | 3.97 | 5.98 | 0.32 | 13.97 | 20.12 | 3.04 | 5.19 | 0.02 | 100 |
| Spectrum11 | 2 | 58.89 | 2.15 | 1.63 | 16.92 | 0.48 | 0.86 | 6.14 | 4.76 | 7.45 | 0.72 | 100 |
| Spectrum12 | 2 | 59.93 | 1.14 | 3.23 | 17.64 | 0.45 | 0.87 | 5.02 | 4.21 | 7.51 | 0.00 | 100 |
| Spectrum13 | 2 | 59.99 | 0.62 | 2.31 | 17.43 | 0.51 | 0.71 | 5.51 | 5.01 | 7.45 | 0.46 | 100 |
| Spectrum14 | 2 | 60.34 | 1.43 | 1.20 | 16.34 | 0.54 | 0.61 | 5.81 | 5.34 | 8.05 | 0.34 | 100 |
| Spectrum15 | 2 | 60.87 | 1.08 | 9.86 | 6.75 | 0.63 | 1.16 | 6.83 | 10.21 | 2.50 | 0.11 | 100 |
| Spectrum16 | 2 | 60.01 | 1.74 | 8.99 | 5.76 | 0.69 | 1.16 | 7.28 | 12.12 | 1.84 | 0.41 | 100 |
| Spectrum17 | 2 | 59.32 | 1.74 | 8.48 | 5.98 | 0.00 | 1.12 | 7.01 | 14.37 | 1.98 | 0.00 | 100 |
| Spectrum18 | 2 | 59.98 | 1.73 | 8.69 | 6.32 | 0.53 | 2.08 | 7.12 | 10.31 | 3.01 | 0.23 | 100 |
| Spectrum19 | 2 | 60.54 | 1.67 | 8.98 | 6.12 | 0.42 | 1.20 | 6.98 | 10.12 | 3.97 | 0.00 | 100 |
| Spectrum20 | 2 | 59.84 | 1.41 | 9.12 | 6.78 | 0.23 | 1.01 | 6.89 | 10.09 | 3.87 | 0.76 | 100 |
| Spectrum21 | 3 | 41.23 | 3.12 | 8.56 | 23.46 | 1.10 | 5.43 | 11.21 | 3.11 | 2.12 | 0.66 | 100 |
| Spectrum22 | 3 | 41.32 | 3.16 | 8.97 | 23.78 | 1.32 | 4.39 | 11.54 | 3.01 | 2.42 | 0.09 | 100 |
| Spectrum23 | 3 | 41.01 | 3.06 | 8.92 | 23.98 | 0.03 | 4.98 | 12.87 | 2.71 | 2.41 | 0.03 | 100 |
| Spectrum24 | 3 | 42.05 | 2.97 | 8.78 | 24.34 | 0.03 | 4.66 | 12.65 | 2.39 | 2.04 | 0.09 | 100 |
| Spectrum25 | 3 | 42.45 | 3.12 | 8.90 | 25.75 | 0.06 | 4.01 | 11.62 | 2.39 | 1.67 | 0.03 | 100 |
| Spectrum26 | 3 | 43.53 | 3.09 | 9.34 | 26.43 | 0.00 | 1.98 | 11.34 | 2.17 | 2.06 | 0.06 | 100 |
| Spectrum27 | 3 | 43.56 | 2.79 | 8.44 | 25.12 | 0.00 | 4.76 | 11.21 | 2.16 | 1.87 | 0.09 | 100 |
| Spectrum28 | 3 | 42.45 | 3.08 | 8.57 | 25.34 | 0.35 | 3.65 | 12.43 | 2.06 | 1.76 | 0.31 | 100 |
| Spectrum29 | 3 | 42.48 | 2.89 | 8.97 | 23.43 | 0.00 | 4.87 | 12.67 | 2.02 | 2.31 | 0.36 | 100 |
| Spectrum30 | 3 | 41.01 | 2.86 | 9.05 | 23.12 | 0.11 | 6.43 | 12.87 | 2.00 | 2.01 | 0.54 | 100 |
| Spectrum31 | 3 | 41.09 | 3.01 | 9.21 | 24.54 | 0.12 | 5.31 | 12.43 | 2.24 | 1.97 | 0.08 | 100 |
| Spectrum32 | 3 | 41.14 | 3.06 | 8.98 | 24.31 | 0.32 | 5.32 | 12.21 | 2.46 | 2.04 | 0.16 | 100 |
| Spectrum33 | 4 | 62.98 | 1.94 | 5.63 | 3.50 | 0.15 | 3.50 | 7.99 | 7.94 | 5.31 | 1.07 | 100 |
| Spectrum34 | 4 | 62.58 | 2.06 | 5.60 | 3.71 | 0.16 | 3.71 | 8.48 | 8.06 | 5.43 | 0.21 | 100 |
| Spectrum35 | 4 | 61.19 | 2.22 | 6.23 | 4.01 | 0.17 | 4.01 | 9.17 | 8.22 | 4.46 | 0.32 | 100 |
| Spectrum36 | 4 | 62.34 | 1.91 | 5.56 | 3.45 | 0.15 | 3.45 | 7.89 | 7.91 | 6.50 | 0.84 | 100 |
| Spectrum37 | 4 | 63.12 | 1.87 | 5.44 | 3.38 | 0.14 | 3.38 | 7.72 | 7.87 | 6.50 | 0.58 | 100 |
| Spectrum38 | 4 | 62.14 | 1.94 | 5.64 | 3.51 | 0.15 | 3.51 | 8.01 | 7.94 | 6.76 | 0.40 | 100 |
| Spectrum39 | 4 | 62.75 | 0.56 | 7.44 | 4.62 | 0.20 | 4.62 | 5.56 | 6.56 | 7.50 | 0.30 | 100 |
| Spectrum40 | 4 | 62.53 | 0.74 | 5.12 | 4.94 | 0.21 | 4.94 | 6.28 | 8.45 | 6.50 | 0.30 | 100 |
| Spectrum41 | 4 | 62.24 | 1.03 | 4.50 | 5.46 | 0.23 | 5.46 | 6.47 | 7.99 | 6.50 | 0.12 | 100 |
| Spectrum42 | 4 | 61.98 | 1.08 | 6.04 | 3.75 | 0.16 | 3.75 | 8.58 | 6.76 | 7.50 | 0.40 | 100 |

**Supplement 3. SEM EDS major element compositions for volcanic glasses from the Abrigo and Granadilla ignimbrites, near Tajao, Tenerife.**

**3.1 El Abrigo volcanic glass compositions in wt%**

| Name | SiO_2_ | TiO_2_ | Fe_2_O_3_T | Al_2_O_3_ | MnO | MgO | CaO | Na_2_O | K_2_O | P_2_O_5_ | Total |
| --- | --- | --- | --- | --- | --- | --- | --- | --- | --- | --- | --- |
| Spectrum01 | 56.03 | 3.15 | 10.02 | 13.89 | 0.00 | 2.76 | 1.18 | 7.67 | 5.19 | 0.11 | 100 |
| Spectrum02 | 56.74 | 2.40 | 10.34 | 13.97 | 0.12 | 3.15 | 1.55 | 7.09 | 4.64 | 0.00 | 100 |
| Spectrum03 | 55.71 | 2.78 | 10.66 | 14.02 | 0.15 | 3.16 | 1.12 | 7.31 | 4.91 | 0.16 | 100 |
| Spectrum04 | 55.98 | 2.87 | 10.15 | 14.19 | 0.00 | 2.95 | 1.39 | 7.48 | 4.90 | 0.11 | 100 |
| Spectrum05 | 57.59 | 2.61 | 10.05 | 14.01 | 0.03 | 2.19 | 1.29 | 6.78 | 5.28 | 0.16 | 100 |
| Spectrum06 | 59.45 | 2.01 | 8.52 | 12.94 | 0.00 | 5.22 | 0.61 | 7.80 | 3.45 | 0.00 | 100 |
| Spectrum07 | 59.77 | 2.21 | 8.94 | 12.87 | 0.00 | 5.09 | 0.77 | 6.79 | 3.56 | 0.00 | 100 |
| Spectrum08 | 60.12 | 1.86 | 9.02 | 12.90 | 0.06 | 4.91 | 0.82 | 6.80 | 3.49 | 0.00 | 100 |
| Spectrum09 | 60.08 | 1.94 | 8.90 | 12.92 | 0.03 | 4.69 | 0.81 | 7.31 | 3.31 | 0.00 | 100 |
| Spectrum10 | 59.53 | 2.03 | 9.16 | 12.72 | 0.03 | 5.07 | 0.71 | 7.31 | 3.43 | 0.00 | 100 |
| Spectrum11 | 59.92 | 1.94 | 8.89 | 12.71 | 0.00 | 4.88 | 0.71 | 7.49 | 3.47 | 0.00 | 100 |
| Spectrum12 | 60.21 | 3.23 | 6.49 | 13.20 | 0.13 | 4.81 | 1.35 | 6.02 | 4.56 | 0.00 | 100 |
| Spectrum13 | 59.39 | 3.40 | 6.51 | 13.24 | 0.13 | 5.17 | 1.63 | 5.87 | 4.47 | 0.19 | 100 |
| Spectrum14 | 58.62 | 1.55 | 8.62 | 13.39 | 0.00 | 5.86 | 0.94 | 7.67 | 3.35 | 0.00 | 100 |
| Spectrum15 | 58.00 | 1.67 | 8.63 | 13.40 | 0.00 | 6.00 | 0.86 | 7.66 | 3.66 | 0.10 | 100 |
| Spectrum16 | 57.93 | 1.32 | 8.31 | 13.21 | 0.09 | 6.00 | 1.07 | 8.21 | 3.71 | 0.15 | 100 |
| Spectrum17 | 59.05 | 1.76 | 8.38 | 12.82 | 0.00 | 6.01 | 1.02 | 7.12 | 3.59 | 0.26 | 100 |
| Spectrum18 | 57.45 | 1.50 | 8.71 | 13.53 | 0.15 | 6.20 | 0.92 | 7.86 | 3.59 | 0.10 | 100 |
| Spectrum19 | 59.02 | 1.40 | 8.46 | 12.84 | 0.09 | 6.90 | 0.96 | 6.90 | 3.45 | 0.00 | 100 |
| Spectrum20 | 59.11 | 1.40 | 8.88 | 12.60 | 0.00 | 6.51 | 0.91 | 7.42 | 3.16 | 0.00 | 100 |
| Spectrum21 | 57.27 | 1.67 | 8.88 | 13.39 | 0.12 | 6.55 | 0.91 | 7.84 | 3.38 | 0.00 | 100 |
| Spectrum22 | 59.46 | 1.58 | 8.61 | 12.46 | 0.06 | 6.16 | 0.86 | 7.45 | 3.06 | 0.31 | 100 |
| Spectrum23 | 57.07 | 2.59 | 8.63 | 14.01 | 0.00 | 3.80 | 0.98 | 8.70 | 3.96 | 0.26 | 100 |
| Spectrum24 | 57.24 | 2.37 | 7.26 | 13.58 | 0.00 | 5.24 | 1.01 | 9.11 | 3.78 | 0.41 | 100 |
| Spectrum25 | 58.03 | 2.30 | 8.54 | 13.43 | 0.12 | 4.87 | 1.08 | 7.65 | 3.68 | 0.30 | 100 |
| Spectrum26 | 58.53 | 1.67 | 7.80 | 13.67 | 0.14 | 6.04 | 1.02 | 7.56 | 3.45 | 0.12 | 100 |
| Spectrum27 | 56.04 | 1.84 | 8.24 | 13.32 | 0.08 | 6.86 | 1.09 | 8.56 | 3.86 | 0.11 | 100 |
| Spectrum28 | 56.24 | 1.76 | 9.18 | 13.06 | 0.12 | 6.03 | 0.98 | 8.27 | 4.02 | 0.34 | 100 |
| Spectrum29 | 59.02 | 2.43 | 8.43 | 12.69 | 0.02 | 5.32 | 1.02 | 7.54 | 3.52 | 0.01 | 100 |
| Spectrum30 | 59.51 | 2.41 | 8.76 | 13.50 | 0.32 | 2.54 | 1.21 | 7.43 | 3.98 | 0.34 | 100 |
| Spectrum31 | 58.18 | 2.76 | 8.02 | 13.67 | 0.18 | 4.37 | 0.87 | 7.94 | 3.79 | 0.21 | 100 |
| Spectrum32 | 57.73 | 3.30 | 8.35 | 14.68 | 0.00 | 3.80 | 0.77 | 7.57 | 3.75 | 0.05 | 100 |
| Spectrum33 | 60.87 | 2.43 | 8.75 | 12.59 | 0.21 | 3.45 | 1.01 | 7.21 | 3.32 | 0.16 | 100 |
| Spectrum34 | 60.92 | 2.21 | 7.98 | 13.11 | 0.12 | 3.78 | 0.99 | 7.27 | 3.42 | 0.20 | 100 |
| Spectrum35 | 59.76 | 1.55 | 8.62 | 14.07 | 0.00 | 3.87 | 1.04 | 7.42 | 3.67 | 0.00 | 100 |
| Spectrum36 | 59.72 | 1.86 | 8.58 | 13.57 | 0.06 | 3.69 | 0.96 | 7.34 | 4.12 | 0.10 | 100 |
| Spectrum37 | 60.78 | 1.54 | 9.50 | 13.37 | 0.01 | 3.19 | 1.02 | 6.98 | 3.52 | 0.09 | 100 |
| Spectrum38 | 60.82 | 1.34 | 9.85 | 13.15 | 0.07 | 3.07 | 1.03 | 7.11 | 3.51 | 0.05 | 100 |
| Spectrum39 | 58.54 | 1.23 | 8.43 | 12.82 | 0.00 | 6.12 | 0.78 | 7.43 | 4.65 | 0.00 | 100 |
| Spectrum40 | 58.78 | 1.43 | 8.18 | 12.84 | 0.00 | 6.04 | 0.85 | 7.65 | 4.23 | 0.00 | 100 |
| Spectrum41 | 57.63 | 3.02 | 8.07 | 13.85 | 0.09 | 4.36 | 0.82 | 8.11 | 3.94 | 0.10 | 100 |

**3.**2 Granadilla volcanic glass compositions in wt%

| Name | SiO_2_ | TiO_2_ | Fe_2_O_3_T | Al_2_O_3_ | MnO | MgO | CaO | Na_2_O | K_2_O | P_2_O_5_ | Total |
| --- | --- | --- | --- | --- | --- | --- | --- | --- | --- | --- | --- |
| Spectrum01 | 61.09 | 1.22 | 12.87 | 9.25 | 0.00 | 1.74 | 0.12 | 0.63 | 13.09 | 0.00 | 100 |
| Spectrum02 | 59.50 | 1.54 | 13.79 | 5.89 | 0.00 | 2.13 | 1.65 | 4.75 | 10.75 | 0.00 | 100 |
| Spectrum03 | 60.21 | 0.94 | 14.08 | 8.23 | 0.00 | 2.91 | 0.51 | 3.95 | 9.19 | 0.00 | 100 |
| Spectrum04 | 59.53 | 0.87 | 13.87 | 6.44 | 0.00 | 2.31 | 1.43 | 4.32 | 11.23 | 0.00 | 100 |
| Spectrum05 | 60.64 | 1.63 | 12.33 | 9.37 | 0.00 | 0.94 | 1.71 | 2.65 | 10.72 | 0.00 | 100 |
| Spectrum06 | 59.78 | 0.32 | 12.31 | 8.92 | 0.00 | 2.65 | 0.43 | 4.98 | 10.61 | 0.00 | 100 |
| Spectrum07 | 64.64 | 2.93 | 12.32 | 5.21 | 0.07 | 2.64 | 0.00 | 2.31 | 9.89 | 0.00 | 100 |
| Spectrum08 | 63.08 | 1.32 | 12.48 | 6.62 | 0.07 | 3.22 | 1.31 | 2.81 | 9.10 | 0.00 | 100 |
| Spectrum09 | 60.87 | 0.85 | 15.23 | 6.14 | 0.00 | 0.84 | 1.77 | 4.14 | 10.15 | 0.00 | 100 |
| Spectrum10 | 62.26 | 0.93 | 16.17 | 4.63 | 0.00 | 1.32 | 0.63 | 2.04 | 12.02 | 0.00 | 100 |
| Spectrum11 | 63.67 | 0.38 | 12.40 | 4.73 | 0.07 | 2.31 | 2.04 | 0.88 | 13.16 | 0.37 | 100 |
| Spectrum12 | 62.33 | 0.13 | 12.56 | 10.12 | 0.07 | 1.91 | 1.00 | 1.49 | 10.10 | 0.30 | 100 |
| Spectrum13 | 63.07 | 0.00 | 13.33 | 6.32 | 0.00 | 1.51 | 0.17 | 3.17 | 12.14 | 0.28 | 100 |
| Spectrum14 | 60.37 | 0.87 | 13.64 | 7.65 | 0.00 | 1.34 | 0.23 | 4.56 | 11.34 | 0.00 | 100 |
| Spectrum15 | 64.18 | 1.00 | 8.72 | 12.91 | 0.00 | 0.62 | 0.29 | 4.42 | 7.87 | 0.00 | 100 |
| Spectrum16 | 61.88 | 0.52 | 9.67 | 7.93 | 0.07 | 2.29 | 1.30 | 4.74 | 11.61 | 0.00 | 100 |
| Spectrum17 | 62.67 | 0.96 | 10.63 | 7.17 | 0.13 | 2.15 | 0.38 | 4.47 | 11.44 | 0.00 | 100 |
| Spectrum18 | 61.17 | 0.31 | 12.30 | 11.65 | 0.00 | 0.31 | 0.47 | 3.02 | 10.77 | 0.00 | 100 |
| Spectrum19 | 61.19 | 0.00 | 11.79 | 7.51 | 0.13 | 3.59 | 1.20 | 3.32 | 11.27 | 0.00 | 100 |
| Spectrum20 | 67.75 | 0.87 | 13.36 | 2.37 | 0.00 | 2.68 | 0.91 | 2.87 | 9.10 | 0.09 | 100 |
| Spectrum21 | 61.22 | 1.22 | 12.88 | 5.68 | 0.07 | 3.71 | 0.73 | 5.59 | 8.90 | 0.00 | 100 |
| Spectrum22 | 60.89 | 0.83 | 13.85 | 5.03 | 0.13 | 3.69 | 0.73 | 5.91 | 8.65 | 0.28 | 100 |
| Spectrum23 | 66.63 | 0.00 | 13.32 | 5.02 | 0.07 | 1.80 | 1.19 | 3.43 | 8.36 | 0.19 | 100 |
| Spectrum24 | 61.85 | 0.00 | 13.78 | 4.63 | 0.00 | 3.44 | 2.17 | 3.90 | 9.50 | 0.75 | 100 |
| Spectrum25 | 65.52 | 1.08 | 12.39 | 5.52 | 0.13 | 3.14 | 2.11 | 1.95 | 8.16 | 0.00 | 100 |
| Spectrum26 | 64.17 | 0.00 | 12.77 | 7.57 | 0.13 | 1.66 | 0.88 | 2.31 | 10.50 | 0.00 | 100 |
| Spectrum27 | 65.63 | 0.87 | 13.90 | 4.11 | 0.13 | 2.88 | 0.22 | 2.87 | 9.39 | 0.00 | 100 |
| Spectrum28 | 62.57 | 0.90 | 12.58 | 6.98 | 0.20 | 1.95 | 1.45 | 3.60 | 9.20 | 0.57 | 100 |
| Spectrum29 | 60.76 | 0.12 | 14.12 | 6.70 | 0.19 | 4.25 | 0.06 | 3.55 | 10.25 | 0.00 | 100 |
| Spectrum30 | 63.03 | 1.51 | 14.80 | 5.03 | 0.07 | 3.01 | 0.00 | 3.16 | 9.39 | 0.00 | 100 |
| Spectrum31 | 63.55 | 0.55 | 15.39 | 4.09 | 0.06 | 3.27 | 1.45 | 3.47 | 8.16 | 0.00 | 100 |
| Spectrum32 | 62.02 | 0.75 | 16.05 | 6.15 | 0.00 | 1.40 | 0.57 | 3.51 | 9.27 | 0.28 | 100 |
| Spectrum33 | 61.73 | 0.87 | 15.48 | 5.11 | 0.00 | 3.08 | 0.17 | 4.70 | 8.21 | 0.64 | 100 |
| Spectrum34 | 61.20 | 1.11 | 16.09 | 2.35 | 0.06 | 2.67 | 0.89 | 3.29 | 11.42 | 0.91 | 100 |
| Spectrum35 | 66.79 | 1.53 | 14.91 | 2.75 | 0.13 | 1.76 | 0.28 | 2.05 | 9.82 | 0.00 | 100 |
| Spectrum36 | 60.96 | 0.00 | 12.76 | 8.04 | 0.00 | 2.67 | 0.29 | 2.88 | 11.46 | 0.95 | 100 |
| Spectrum37 | 63.28 | 1.07 | 16.73 | 3.78 | 0.00 | 1.81 | 1.36 | 2.46 | 9.51 | 0.00 | 100 |
| Spectrum38 | 62.00 | 0.99 | 15.11 | 4.64 | 0.00 | 1.64 | 0.32 | 2.54 | 12.75 | 0.00 | 100 |
| Spectrum39 | 63.50 | 0.99 | 13.09 | 5.42 | 0.00 | 1.67 | 0.32 | 2.65 | 12.35 | 0.00 | 100 |
| Spectrum40 | 63.50 | 0.68 | 14.33 | 4.32 | 0.00 | 2.04 | 0.23 | 2.65 | 12.24 | 0.00 | 100 |
| Spectrum41 | 65.50 | 1.02 | 15.02 | 2.42 | 0.00 | 1.37 | 0.98 | 4.01 | 9.23 | 0.45 | 100 |
| Spectrum39 | 65.80 | 1.05 | 17.11 | 2.51 | 0.00 | 1.32 | 0.87 | 2.40 | 8.42 | 0.52 | 100 |
| Spectrum40 | 60.45 | 0.96 | 15.44 | 3.15 | 0.00 | 3.23 | 1.32 | 3.54 | 11.91 | 0.00 | 100 |
| Spectrum41 | 62.46 | 0.00 | 15.73 | 0.87 | 0.00 | 1.28 | 1.90 | 3.46 | 12.11 | 2.19 | 100 |
| Spectrum42 | 61.21 | 0.19 | 15.85 | 3.07 | 0.00 | 0.50 | 1.36 | 4.21 | 11.38 | 2.24 | 100 |
| Spectrum43 | 62.57 | 0.00 | 16.27 | 3.38 | 0.00 | 1.63 | 0.80 | 5.43 | 9.73 | 0.19 | 100 |
| Spectrum44 | 65.90 | 0.00 | 12.59 | 4.17 | 0.13 | 1.37 | 0.88 | 5.14 | 9.46 | 0.36 | 100 |
| Spectrum45 | 60.67 | 0.87 | 16.54 | 4.02 | 0.00 | 1.98 | 0.73 | 4.45 | 10.74 | 0.00 | 100 |
| Spectrum46 | 60.50 | 0.31 | 18.10 | 3.38 | 0.07 | 2.00 | 0.85 | 4.46 | 10.35 | 0.00 | 100 |
| Spectrum47 | 61.42 | 0.13 | 16.61 | 4.80 | 0.00 | 1.31 | 1.14 | 4.85 | 9.75 | 0.00 | 100 |

**Supplement 4. SEM EDS major element compositions of international standard reference materials in wt%.**

| Standard | JB3 |  |  |  |  |  |  |  |  |  |
| --- | --- | --- | --- | --- | --- | --- | --- | --- | --- | --- |
| # | Spectrum01 | Spectrum02 | Spectrum03 | Spectrum04 | Spectrum05 | Av | StDev | % StDev* | Certified | % Error* |
| SiO2 | 51.61 | 50.92 | 51.45 | 51.24 | 51.39 | 51.32 | 0.26 | 0.5% | 50.96 | 0.7% |
| TiO2 | 0.96 | 1.03 | 1.11 | 1.05 | 1.05 | 1.04 | 0.05 | 5.0% | 1.44 | 38.3% |
| Al2O3 | 18.78 | 18.96 | 18.03 | 18.50 | 18.68 | 18.59 | 0.35 | 1.9% | 17.20 | 7.5% |
| Fe2O3 | 9.89 | 10.37 | 10.48 | 10.21 | 10.47 | 10.28 | 0.25 | 2.4% | 11.82 | 14.9% |
| MnO | 0.16 | 0.47 | 0.31 | 0.31 | 0.39 | 0.33 | 0.12 | 35.2% | 0.18 | 45.9% |
| MgO | 4.98 | 5.02 | 4.97 | 5.10 | 4.92 | 5.00 | 0.07 | 1.3% | 5.19 | 3.8% |
| CaO | 9.60 | 9.20 | 9.26 | 9.46 | 9.26 | 9.36 | 0.17 | 1.8% | 9.79 | 4.6% |
| Na2O | 2.81 | 2.98 | 2.95 | 2.92 | 2.76 | 2.88 | 0.10 | 3.4% | 2.73 | 5.3% |
| K2O | 0.88 | 0.83 | 0.89 | 0.97 | 0.86 | 0.89 | 0.05 | 5.7% | 0.78 | 11.9% |
| P2O5 | 0.34 | 0.22 | 0.55 | 0.25 | 0.22 | 0.32 | 0.14 | 44.4% | 0.29 | 7.0% |
| Total | 100 | 100 | 100 | 100 | 100 |  |  |  |  |  |

| Standard | JB2 |  |  |  |  |  |  |  |  |  |
| --- | --- | --- | --- | --- | --- | --- | --- | --- | --- | --- |
| # | Spectrum01 | Spectrum02 | Spectrum03 | Spectrum04 | Spectrum05 | Av | StDev | % StDev* | Certified | % Error* |
| SiO2 | 53.27 | 52.29 | 52.65 | 51.43 | 53.32 | 52.59 | 0.78 | 1.5% | 53.25 | 1.3% |
| TiO2 | 0.99 | 1.20 | 1.16 | 1.16 | 1.01 | 1.10 | 0.10 | 8.7% | 1.19 | 7.8% |
| Al2O3 | 15.28 | 14.71 | 14.79 | 14.75 | 14.95 | 14.90 | 0.23 | 1.6% | 14.64 | 1.7% |
| Fe2O3 | 13.73 | 15.11 | 14.11 | 14.93 | 14.11 | 14.40 | 0.59 | 4.1% | 14.25 | 1.0% |
| MnO | 0.16 | 0.23 | 0.16 | 0.24 | 0.16 | 0.19 | 0.04 | 22.8% | 0.22 | 13.8% |
| MgO | 4.57 | 4.53 | 4.65 | 4.68 | 4.51 | 4.59 | 0.08 | 1.7% | 4.62 | 0.7% |
| CaO | 9.31 | 9.48 | 9.59 | 9.81 | 9.54 | 9.55 | 0.18 | 1.9% | 9.82 | 2.9% |
| Na2O | 2.08 | 1.97 | 2.25 | 2.14 | 1.90 | 2.07 | 0.14 | 6.7% | 2.04 | 1.5% |
| K2O | 0.40 | 0.33 | 0.40 | 0.33 | 0.28 | 0.35 | 0.05 | 14.9% | 0.42 | 20.0% |
| P2O5 | 0.22 | 0.34 | 0.23 | 0.23 | 0.23 | 0.25 | 0.05 | 20.7% | 0.10 | 59.7% |
| Total | 100 | 100 | 100 | 100 | 100 |  |  |  |  |  |

| Standard | JB1a |  |  |  |  |  |  |  |  |  |
| --- | --- | --- | --- | --- | --- | --- | --- | --- | --- | --- |
| # | Spectrum01 | Spectrum02 | Spectrum03 | Spectrum04 | Spectrum05 | Av | StDev | % StDev* | Certified | % Error* |
| SiO2 | 53.48 | 53.94 | 53.61 | 54.52 | 53.90 | 53.89 | 0.40 | 0.7% | 52.41 | 2.8% |
| TiO2 | 1.22 | 1.14 | 1.16 | 1.06 | 1.29 | 1.17 | 0.09 | 7.5% | 1.28 | 9.1% |
| Al2O3 | 15.33 | 15.52 | 16.06 | 15.50 | 15.42 | 15.56 | 0.29 | 1.8% | 14.45 | 7.2% |
| Fe2O3 | 8.18 | 8.05 | 8.18 | 7.50 | 7.77 | 7.94 | 0.30 | 3.7% | 9.05 | 14.0% |
| MnO | 0.16 | 0.39 | 0.16 | 0.47 | 0.32 | 0.30 | 0.14 | 46.5% | 0.15 | 50.5% |
| MgO | 7.82 | 7.65 | 7.65 | 7.71 | 7.62 | 7.69 | 0.08 | 1.0% | 7.83 | 1.8% |
| CaO | 9.24 | 8.89 | 8.71 | 8.48 | 9.27 | 8.92 | 0.34 | 3.8% | 9.31 | 4.4% |
| Na2O | 3.02 | 2.87 | 2.83 | 2.99 | 2.82 | 2.91 | 0.09 | 3.2% | 2.73 | 6.1% |
| K2O | 1.44 | 1.43 | 1.53 | 1.21 | 1.48 | 1.42 | 0.12 | 8.7% | 1.40 | 1.4% |
| P2O5 | 0.11 | 0.11 | 0.11 | 0.55 | 0.11 | 0.20 | 0.20 | 98.4% | 0.26 | 29.6% |
| Total | 100 | 100 | 100 | 100 | 100 |  |  |  |  |  |

| Standard | UMAT-1 |  |  |  |  |  |  |  |  |  |
| --- | --- | --- | --- | --- | --- | --- | --- | --- | --- | --- |
| # | Spectrum01 | Spectrum02 | Spectrum03 | Spectrum04 | Spectrum05 | Av | StDev | % StDev* | Certified | % Error* |
| SiO2 | 53.19 | 53.32 | 53.14 | 53.55 | 53.84 | 53.41 | 0.29 | 0.5% | 53.57 | 0.3% |
| TiO2 | 3.01 | 3.11 | 3.25 | 3.02 | 2.95 | 3.07 | 0.12 | 3.8% | 2.79 | 9.1% |
| Al2O3 | 13.83 | 13.11 | 13.35 | 13.21 | 13.25 | 13.35 | 0.28 | 2.1% | 13.48 | 1.0% |
| Fe2O3 | 13.35 | 13.21 | 13.43 | 13.22 | 13.06 | 13.25 | 0.14 | 1.1% | 12.54 | 5.4% |
| MnO | 0.41 | 0.32 | 0.25 | 0.56 | 0.40 | 0.39 | 0.12 | 30.2% | 0.21 | 45.8% |
| MgO | 2.65 | 2.88 | 2.82 | 2.91 | 2.77 | 2.81 | 0.10 | 3.7% | 2.87 | 2.3% |
| CaO | 7.33 | 7.14 | 6.92 | 6.92 | 7.07 | 7.08 | 0.17 | 2.4% | 6.39 | 9.7% |
| Na2O | 3.52 | 3.60 | 3.69 | 3.82 | 3.57 | 3.64 | 0.12 | 3.2% | 3.25 | 10.8% |
| K2O | 2.60 | 2.85 | 2.70 | 2.44 | 2.40 | 2.60 | 0.18 | 7.1% | 2.60 | 0.0% |
| P2O5 | 0.12 | 0.46 | 0.45 | 0.34 | 0.68 | 0.41 | 0.20 | 49.9% | 0.89 | 115.8% |
| Total | 100 | 100 | 100 | 100 | 100 |  |  |  |  |  |

| Standard | JGb-1 |  |  |  |  |  |  |  |  |  |
| --- | --- | --- | --- | --- | --- | --- | --- | --- | --- | --- |
| # | Spectrum01 | Spectrum02 | Spectrum03 | Spectrum04 | Spectrum05 | Av | StDev | % StDev* | Certified | % Error* |
| SiO2 | 45.59 | 45.75 | 45.61 | 44.58 | 45.17 | 45.34 | 0.48 | 1.1% | 43.66 | 3.8% |
| TiO2 | 1.55 | 1.43 | 1.72 | 1.57 | 1.44 | 1.54 | 0.12 | 7.6% | 1.60 | 3.6% |
| Al2O3 | 18.24 | 18.27 | 18.33 | 19.05 | 18.70 | 18.52 | 0.35 | 1.9% | 17.45 | 6.1% |
| Fe2O3 | 13.59 | 13.53 | 13.77 | 13.85 | 13.89 | 13.72 | 0.16 | 1.2% | 15.06 | 8.9% |
| MnO | 0.16 | 0.24 | 0.24 | 0.40 | 0.16 | 0.24 | 0.10 | 41.0% | 0.19 | 28.2% |
| MgO | 7.72 | 8.00 | 7.62 | 7.66 | 7.64 | 7.73 | 0.16 | 2.0% | 7.85 | 1.5% |
| CaO | 11.62 | 11.03 | 11.04 | 11.05 | 11.17 | 11.18 | 0.25 | 2.2% | 11.90 | 6.0% |
| Na2O | 1.20 | 1.17 | 0.93 | 1.21 | 1.16 | 1.13 | 0.12 | 10.2% | 1.20 | 5.5% |
| K2O | 0.22 | 0.22 | 0.29 | 0.28 | 0.22 | 0.25 | 0.03 | 14.0% | 0.24 | 2.7% |
| P2O5 | 0.11 | 0.34 | 0.44 | 0.34 | 0.45 | 0.34 | 0.14 | 40.3% | 0.06 | 504.7% |
| Total | 100 | 100 | 100 | 100 | 100 |  |  |  |  |  |
